# Delineating the dynamic evolution from preneoplasia to invasive lung adenocarcinoma by integrating single-cell RNA sequencing and spatial transcriptomics

**DOI:** 10.1038/s12276-022-00896-9

**Published:** 2022-11-25

**Authors:** Jianfei Zhu, Yue Fan, Yanlu Xiong, Wenchen Wang, Jiakuan Chen, Yanmin Xia, Jie Lei, Li Gong, Shiquan Sun, Tao Jiang

**Affiliations:** 1 Department of Thoracic Surgery, Tangdu Hospital, Air Force Medical University (Fourth Military Medical University), Xi’an, Shaanxi PR China; 2 Department of Thoracic Surgery, Shaanxi Provincial People’s Hospital, Xi’an, Shaanxi PR China; 3 School of Public Health, Xi’an Jiaotong University Health Science Center, Xi’an, Shaanxi PR China; 4 Center for Single Cell Omics and Health, Key Laboratory of Trace Elements and Endemic Diseases, Xi’an Jiaotong University, Xi’an, Shaanxi PR China; 5 Department of Pathology, Tangdu Hospital, Air Force Medical University (Fourth Military Medical University), Xi’an, Shaanxi PR China

**Keywords:** Non-small-cell lung cancer, Cancer microenvironment

## Abstract

The cell ecology and spatial niche implicated in the dynamic and sequential process of lung adenocarcinoma (LUAD) from adenocarcinoma in situ (AIS) to minimally invasive adenocarcinoma (MIA) and subsequent invasive adenocarcinoma (IAC) have not yet been elucidated. Here, we performed an integrative analysis of single-cell RNA sequencing (scRNA-seq) and spatial transcriptomics (ST) to characterize the cell atlas of the invasion trajectory of LUAD. We found that the UBE2C + cancer cell subpopulation constantly increased during the invasive process of LUAD with remarkable elevation in IAC, and its spatial distribution was in the peripheral cancer region of the IAC, representing a more malignant phenotype. Furthermore, analysis of the TME cell type subpopulation showed a constant decrease in mast cells, monocytes, and lymphatic endothelial cells, which were implicated in the whole process of invasive LUAD, accompanied by an increase in NK cells and MALT B cells from AIS to MIA and an increase in Tregs and secretory B cells from MIA to IAC. Notably, for AIS, cancer cells, NK cells, and mast cells were colocalized in the cancer region; however, for IAC, Tregs colocalized with cancer cells. Finally, communication and interaction between cancer cells and TME cell-induced constitutive activation of TGF-β signaling were involved in the invasion of IAC. Therefore, our results reveal the specific cellular information and spatial architecture of cancer cells and TME subpopulations, as well as the cellular interaction between them, which will facilitate the identification and development of precision medicine in the invasive process of LUAD from AIS to IAC.

## Lung cancer: Mapping the invasion

A map showing how lung cancer progresses from static to invasive carcinomas could inform new therapeutic approaches. The World Health Organisation defines three stages of lung adenocarcinoma: first, in situ lesions, then minimally invasive, then fully invasive. Details about the cell populations involved and their locations during cancer development have been limited. Jianfei Zhu at Air Force Medical University in Xi’an, China, and co-workers have combined the strengths of single-cell RNA sequencing and spatial transcriptomics to produce a map showing where and when different cell types become involved in lung cancer progression. They showed that UBE2C cells, well-known tumor-forming cells, are involved at early stages, and could be key clinical indicators of invasion. Later, regulatory T-cells, which can suppress the body’s immune responses to tumors, are recruited into cancer regions while other cell types are reduced.

## Introduction

In the latest lung cancer classification system by the WHO (5^th^ edition)^1^, lung adenocarcinoma in situ (AIS), mainly identified as a ground-glass nodule (GGN) as a precursor glandular lesion, was excluded from the lung adenocarcinoma (LUAD) category due to its substantial difference from minimally invasive adenocarcinoma (MIA) and invasive adenocarcinoma (IAC) in long-term outcomes^2,3^. However, the detailed cell population and genes, as well as their spatial information implicated in the invasive progression of LUAD from AIS to IAC, remain poorly understood.

Canonical cancer gene mutations in cancer cells, such as EGFR and TP53, have been widely reported in AIS, which promoted the invasion of LUAD from AIS to IAC by the accumulation of mutations in the branch of the evolutionary tree, potentially “switching” molecules responsible for invasive events^4–7^. Moreover, a pioneering study revealed that dysregulation of immune surveillance of the tumor microenvironment (TME) occurs through the process of invasive LUAD, even in AIS^4^. Although the genomic landscape of preinvasive LUAD (AIS and MIA) has been clarified to some extent using the traditional sequencing strategy, such as bulk whole-exome sequencing (WES) and bulk RNA sequencing (bulk RNA-seq)^5,6^, a mixed population of cancer cells and TME components in one tissue was generally used for in silico analysis in this scenario. Therefore, sequencing technology to determine cell-type-specific profiles is urgently needed.

With the advent of single-cell RNA sequencing (scRNA-seq), it has been regarded as an unprecedented tool for dissecting the heterogeneity of cancer at the single-cell level and developing a comprehensive gene expression atlas. Studies on the preinvasive LUAD genomic atlas determined by scRNA-seq are limited, and only a few studies have decoded the multicellular environment of GGNs using this novel technology^8–10^. At the single-cell level, the above studies demonstrate that the enrichment of Tregs and the reduction of cytotoxic CD8 + T cells in the TME are involved in the malignant progression from GGN to IAC. Notably, single-cell dissociation lacks spatial information, which is indispensable for a comprehensive understanding of the pathophysiology and progression of LUAD, especially in its immunophenotyping^11–13^. To address this issue, spatial transcriptomics (ST) seamlessly overcomes the disadvantages of scRNA-seq^14^ and has been widely applied in the study of the spatial distribution of cancer cells and TMEs^15–17^. Since the strengths of scRNA-seq and ST are complementary, combining these two technologies can simultaneously reveal the heterogeneity of cancers and the spatial distribution of their diverse ecosystems. However, such multiomics data have not been elucidated in the invasive process of LUAD.

To illuminate the evolutionary trajectory of LUAD, high-throughput scRNA-seq and ST data were generated and integrated to create a large-scale, single-cell spatiotemporal multiomics atlas of invasive LUAD to reflect the heterogeneity of cancer tissues, distinct cancer cells, and TME cell populations in LUAD with different invasive states, as well as the signaling interactions between cancer cells and the TME from cellular and spatial perspectives from AIS to IAC. The advent of this illuminating study will provide strong theoretical evidence to improve the clinical diagnosis and surgical intervention of early-stage LUAD.

## Materials and methods

### Patient enrollment and specimen collection

Nine specimens were collected from nine patients with primary LUAD who were first examined and underwent surgery in the Department of Thoracic Surgery, Tangdu Hospital (Supplementary Table 1). The inclusion criteria were as follows: (1) single lung nodule; (2) maximum diameter of the tumor ≤ 3.0 cm; (3) histological diagnosis of LUAD; (4) no previous history of other malignant tumors; (5) negative pathological N stage; (6) no distant metastasis; and (6) radical lobectomy/segmentectomy. The pathological diagnosis was independently carried out by three experienced pathologists in accordance with the 5th edition of the WHO lung tumor classification system.

### Preparation of single-cell suspensions

Following isolation and pathological clearance of fresh specimens, the tumor samples were rinsed with cold phosphate-buffered saline (PBS). According to preoperative CT axial localization, each tumor sample was divided into two portions along the long axis: one portion was used for scRNA-seq and ST, and the other portion was used for routine and independent pathological diagnosis by two experienced pathologists. Each sample was cut into 3-mm-thick slices along the maximum diameter, placed into optimal cutting temperature (OCT) compound and then frozen in isopentane cooled in liquid nitrogen for further ST; the remaining sample was cut into portions less than 1 mm^3^ in volume. A human tumor dissociation kit enzyme solution (Miltenyi Biotec; 200 µl of H-enzyme, 100 µl of R-enzyme, 25 µl of A-enzyme, 4.7 ml of DMEM) was added for enzymatic digestion for 30 min at 37 °C, and the sample was filtered through a Miltenyi 70-μm sieve. After centrifugation, the granular cells were suspended in erythrocyte lysis buffer. Finally, the cells were mixed with 1 ml of PBS, and the numbers of live cells and aggregated cells were measured with an automatic cell counter^18^.

### scRNA-seq

For droplet-based scRNA-seq, GemCodeGel beads, chips and library kits (10′ Genomics) were used to process single cells according to the gel beads in emulsion (GEMs) protocol (Genergy Inc., Shanghai, China). Based on the manufacturer’s instructions, the Chromium Single Cell 3′ V2/V3 Kit was used to construct the scRNA-seq reagent library. Then, the cell suspension generated from each sample was reacted with certain reagents to generate single-cell GEMs in the Chromium Controller for sample and cell barcoding, with a target output of 8,000 to 12,000 cells per sample. The amplified cDNA and final library were evaluated with an Agilent Bioanalyzer using a high-sensitivity DNA kit (2100, Agilent Technologies). Then, the samples were sequenced using NovaSeq 6000 (Illumina) platforms. Approximately 400 M readings were obtained for each sample.

### scRNA-seq data quality control and integration

ScRNA-seq reads were demultiplexed and aligned to the ENSEMBL GRCh38 human transcriptome to generate gene expression matrices using CellRanger (v4.0.0). The following criteria were then applied to select cells for all nine patients: (1) gene numbers between 200 and 10,000; (2) unique molecular identifier (UMI) count>500; (3) mitochondrial gene percentage < 0.1; and (4) hemoglobin gene percentage <0.1. After filtering, the final dataset consisted of data from 115,246 cells. We then performed integrated analyses using Seurat (version 3.2.2)^19^. Briefly, the filtered gene expression matrix was first normalized using LogNormalize methods, and the top 2000 highly variable features were then selected using the vst method^20^. Next, we identified “anchors” between individual datasets and integrated scRNA-seq data and used these anchors to harmonize the datasets. Finally, we obtained a batch-corrected expression matrix of all cells for downstream analysis.

### Clustering and cell type annotation

The integrated data were scaled, and dimensionality reduction was performed by principal component analysis (PCA). The Louvain clustering algorithm implemented in Seurat was then applied to the PCA reduced data for clustering analysis with 30 principal components (PCs)^21^. The resolution was set to 0.5 to obtain the clustering results. The top 50 PCs were selected for uniform manifold approximation and projection (UMAP) to visualize the cell clustering results^22^. Differentially expressed genes (DEGs) for each cluster were then identified using the Wilcoxon test. Only genes for which the difference in expression had an adjusted *P* value < 0.05 and a logFC > 0.25 were considered marker genes. Clusters were then annotated based on the marker genes of particular cell types. We then performed subclustering for each major cell type. Briefly, data from cells of each major cell type were extracted from the integrated dataset, and clustering was performed as described above with cell type-specific resolution.

### Reproducibility of single-cell studies

We quantified the reproducibility of subclusters for each major cell type through single-cell reproducibility across donors (scRAD)^23^ to assess the important marker gene expression differences across different patients for each condition (AIS, MIA, IAC). Briefly, we first pooled the normalized log-TPM data for each patient and assessed signal reproducibility across 3 patient donors for each condition through irreducible discovery rate (IDR) analysis to emphasize differential expression that is reproducible across patients^24^. For each gene, we can estimate the probability that the gene is an “irreproducible gene” (IDR < = 0.01) or a “reproducible gene” (IDR > 0.01).

### Identification of cancer cells

To identify cancer cells, we searched for copy number variations (CNVs) for each subcluster of the epithelial cell cluster from the scRNA-seq data with inferCNV (version 1.2.1)^25^. Raw count data were extracted from the Seurat object as recommended in the “Using 10× data” section. Endothelial cells were considered reference cells, and their CNV estimates were used to define a baseline. We created a gene ordering file from the human CRCh38 assembly, which contains the chromosomal start and end positions for each gene. For the inferCNV analysis, we used a cutoff of 0.1 for the minimum average read counts per gene among reference cells and denoised the output to predict the CNV level.

### Gene set variation analysis (GSVA)

Gene set enrichment analyses were performed with 50 hallmark pathways that were exported using the Molecular Signatures Database (MSigDB)^26^. To assign pathway activity estimates to each cell type, we performed GSVA for each cell and then calculated the average gene expression level for each cell subcluster and applied GSVA using standard settings with the GSVA package (version 1.34.0)^27^. Differences between activity scores were used to quantify differential pathway activity between different subclusters of cells.

### Prediction of regulons

Single-cell regulatory network inference and clustering (SCENIC) (version 1.2.4) was used to assess the regulatory network consisting of transcription factors (TFs) and discover regulons (TFs and their target genes) for each cell^28^. Following the latest SCENIC pipeline, the integrated gene expression matrix with gene names in rows and cells in columns was input into SCENIC. A human-specific genome (hg19) was input into the RcisTarget database. Briefly, coexpression modules between TFs and potential target genes were identified with GENIE3 (version 1.12.0)^29^, regulons were identified through *cis*-regulatory motif enrichment analysis of all potential target genes with RcisTarget (version 1.10.0), and the activity score of each regulon for each cell was assessed with AUCell (version 1.13.3). To assess differences in regulon activity between subpopulations of cells, we assessed the activity scores for each cell using the Limma package (version 3.42.2)^30^. Differential regulon activities were calculated for each subcluster, and t values were used to quantify these differences.

### Prediction of cancer cell trajectory

We performed trajectory analysis with Monocle2 (version 2.14.0)^31–33^ to characterize the process of cancer cell development and determine lineage differentiation among diverse cancer cells. Following a standard protocol, we directly imported the integrated gene expression data into Monocle2 and then ordered cells based on the DEGs between cancer subclusters according to the significant genes (*q* value<0.01). The cancer cell differentiation trajectory was inferred after dimension reduction with the DDRTree method and cell ordering with the default parameters of Monocle2.

### Cell‒cell communication analysis

We assessed differences in putative cell‒cell communication modules between cell types for samples at each stage (AIS, MIA and IAC) by integrating the gene expression data through CellChat (version 1.0.0)^34^. Following the standard CellChat pipeline, we used the default CellChatDB as the ligand‒receptor database and then inferred cell type-specific communication by identifying overexpressed ligands or receptors in one cell group and then identifying enhanced ligand‒receptor interactions when either the ligand or receptor was overexpressed. We then used NicheNet to investigate the signaling mediators involved in ligand‒receptor pairing among cancer cells signaling with immune cells, endothelial cells, and fibroblasts^35^. During the NicheNet runs, cancer cells were set as ‘receiver’ and other cell types as ‘sender’ populations. For both the sender and receiver populations, the genes of the signaling pathway of interest, which were expressed in at least 10% of the cells, were used for downstream analysis. NicheNet analysis was performed based on the vignette to rank potential ligands, infer receptors, and top-predicted target genes of ligands.

### Tissue handling for ST

The prepared frozen sections described above were sliced at a thickness of 10 µm, embedded on frozen Visium tissue optimization slides (3000394, 10× Genomics) and Visium spatial gene expression slides (2000233, 10× Genomics), and stored at −80 °C until use. Afterward, the sections were fixed in frozen methanol and stained according to the Visium Spatial Gene Expression User Guide (CG000239 Rev A, 10× Genomics) or Visium Spatial Tissue Optimization User Guide (CG000238 Rev A, 10× Genomics). The Qiagen RNeasy Mini Kit was applied for RNA extraction and isolation, after which an Agilent 2100 bioanalyzer was used for RNA integrity number (RIN) calculation (RIN ≥ 7). The tissue with meaningful gene expression was permeabilized for 6 min, regarded as the best time for idealizing tissue according to a time course experiment. A cDNA library was established according to the visium spatial gene expression user guide. The cDNA library was sequenced on a HiSeq 3000 system (Illumina) with a sequencing depth of approximately 250‒270 M reads for each sample.

### ST data processing

The 10× Genomics Spatial RNA-seq Visium platform was used to perform spatial transcriptomics experiments. Hematoxylin and eosin (HE)-stained sections were analyzed via 10× Genomics Space Ranger software (Genergy Inc., Shanghai, China). The spatial RNA-seq data were analyzed with the Seurat package^19^ (version 3.2.2). Briefly, for each sample, the gene-spot matrix was normalized by sctransform^20^, and dimensionality reduction was performed by PCA. The Louvain clustering algorithm was then applied to the reduced data for clustering analysis with 30 PCs. The resolution was set to 0.5 to obtain the clustering result. The DEGs for each cluster were then identified using the Wilcoxon test. Only genes whose differential expression had an adjusted *P* value < 0.05 and a logFC > 0.25 were considered marker genes. To determine the cell type that was enriched in each cluster, we queried the significance of the overlap between ST marker genes and scRNA-seq marker genes using Fisher’s exact test, with all genes as the background, to compute the odds ratio and *P* value.

### Cell type decomposition analysis of spatial transcriptome data

Robust cell type decomposition (RCTD) was used to map the cell types found in the reference scRNA-seq dataset to spatial transcriptomic data^36^. Marker genes for each cell type were obtained using the Seurat function FindAllMarkers, whereby only markers with positive log2-transformed fold changes were considered. We then followed the standard RCTD analysis pipeline on the reference and Visium spatial transcriptomics data in doublet mode set to full.

### Spatial trajectory analysis

Patterns of transient gene expression along spatial trajectories were analyzed by Monocle3 (version 1.0.0), which was easily implemented through SPATA (version 0.1.0)^37,38^. Specifically, spots that belonged to either the epithelial region or the cancer region were extracted and ordered along the pseudotime trajectory. Following the SPATA protocol, we switched the spata object to the cds object and ordered spots based on DEGs between subclusters according to the significant genes (q value<0.01). The spatial trajectory was inferred after dimension reduction with the UMAP method. Monocle3 provides a shiny interface to select a root for pseudotime annotation. We randomly selected spots that belonged to the epithelium region as the root.

### Querying spatial transcriptome data with scRNA-seq data using Cell-ID

We performed Cell-ID^39^ to query the gene expression of each spot in spatial transcriptome data to scRNA-seq clustering results. Following the Cell-ID vignette, we first performed dimensionality reduction through multiple correspondence analysis (MCA) for both scRNA-seq data and spatial transcriptome data, extracted the gene signature of the cell types that clustered in scRNA-seq data, and then performed cell type annotation via hypergeometric tests for each spot based on the gene signature of reference scRNA-seq data.

### Bulk RNA-seq

Total RNA was extracted and prepared from LUAD and paired paracancerous tissues (AIS: 20, MIA: 17, IAC: 23, Supplementary Table 3) following the manufacturer’s instructions by using TRIzol (Invitrogen, CA, USA). The total RNA for each sample was > 1 µg as the initial material for RNA sample preparation. The captured mRNA was fragmented, and cDNA was synthesized. Subsequently, the uracil-DNA glycosylase (UDG) enzyme (NEB, catalog number m0280, MA, US) was used to generate a sequencing library following the manufacturer’s instructions. The library was sequenced on the Illumina Novaseq™ 6000 (LC Bio Technology Co., Ltd., Hangzhou, China) platform, and a 150 bp paired-end read was generated. To explore cancer cell and immune cell landscapes in bulk-seq data, single-sample gene set enrichment analysis (ssGSEA) was performed using the R package “gsva” with the aim of assessing the proportion of cells of interest (recorded as ssGSEA scores) in a single sample based on DEGs from scRNA-seq^27^. The Wilcoxon test was used for comparison of numerical data, and the chi-square test and Fisher test were used for constituent ratio analysis. Finally, the impact of different cellular infiltrations on patient prognosis was analyzed using the TCGA-LUAD databases.

### Immunofluorescence (IF) staining

We established a tissue microarray (TMA, 3 mm) consisting of LUAD samples: 14 AIS samples, 18 MIA samples, and 17 IAC samples (Supplementary Table 4). After antigen repair and blocking, the TMA was incubated with specific primary antibodies (EPCAM, Servicebio, GB14078, 1:200; FOXP3, Servicebio, GB11093, 1:200; TPSB2, Novus, NBP2-33551, 1:300; CD79A, Novus, NB100-64347ss, 1:200; CLDN5, Servicebio, GB11290, 1:200; COL1A1, Affinity, AF7001, 1:200; UBE2C, Abcam, ab12290, 1:50; p-SMAD2, Affinity, AF8314, 1:200; SCGB1A1, Servicebio, GB111412, 1:200; PDPN, Affinity, AF3670-SP, 1:200; FCN1, Novus, NBP1-84706, 1:200; GHD, Proteintech, 67538 1:250; PCNA, Servicebio, GB11010, 1:300), incubated with horseradish peroxidase (HRP)-labeled secondary antibodies or fluorophore-labeled secondary antibodies, and finally stained with diaminobenzidine and counterstained with hematoxylin or stained with DAPI. Three independent pathologists distinguished the pathological type for the TMA.

### Cell culture

The human LUAD cell line PC-9 was purchased from the Cell Bank of the Chinese Academy of Sciences (Shanghai). The cells were cultured in RPMI-1640 medium containing 10% fetal bovine serum (FBS) in a humidified incubator at 37 °C with 5% carbon dioxide.

### Cell infection and transfection

The nucleic acid sequence used to construct the siRNAs was UBE2C: 5′-GCAAGAAACCUACUCAAAGTT-3′, and the control siRNA sequence was 5′-UUCUCCGAACGUGUCACGUTT-3′. Control siRNAs (GenePharma, Shanghai, China) and target siRNAs (GenePharma, Shanghai, China) were transfected into PC-9 human LUAD cells using Lipofectamine 2000 (Invitrogen, CA, USA). Moreover, recombinant lentiviruses (Hanheng Biotechnology, Shanghai, China) for UBE2C gene interference were also induced in PC-9 cells to generate stable cell models. When the results of RT qPCR and western blot analyses demonstrated significant inhibition of UBE2C mRNA and protein levels, then the gene interference was considered to be successful.

### RNA isolation and quantitative reverse transcription polymerase chain reaction (qRT‒PCR)

Total RNA was extracted from frozen tissues using TRIzol reagent (Invitrogen, USA). A Revert Aid First-Strand cDNA Synthesis Kit (Thermo Scientific, Vilnius, Lithuania) was used to prepare reverse transcription according to the manufacturer’s protocols. qRT‒PCR was carried out using LightCycler-Fast-Start DNA Master SYBR Green (Roche Diagnostics, Tokyo, Japan). Gene expression was normalized to β-actin. The mRNA levels were expressed as the threshold cycle (CT). The amount of target was measured using the 2^-△△CT^ method. The primers used for qRT‒PCR were UBE2C-F: 5′-AGTGGCTACCCTTACAATGCG-3′ and UBE2C-R: 5′-TTACCCTGGGTGTCCACGTT-3′.

### Transwell experiment

Transwell chambers (8-μm pores; Corning Inc., New York) and an artificial basement membrane (Matrigel) (Corning Inc.) (BD Biosciences, USA) were used to test the ability of cells to invade. A cell suspension in medium without fetal bovine serum was added to the upper compartment, and medium containing 20% fetal bovine serum was added to the lower compartment. After culture for 24 to 48 h, the cells on the lower surface were fixed with methanol and stained with 0.2% crystal violet for counting. An additional Transwell chamber without an artificial basement membrane (Corning) was simultaneously used to test cell migration. Cell counts were used as the mean ± standard error, an unpaired T test was used for comparisons between different groups, and a two-tailed *P* value < 0.05 was considered statistically significant.

### Cell proliferation assay

When cells were in the logarithmic growth phase, they were trypsinized and diluted to 6.5 × 10^4^ cells/mL. The CIM detection plate dedicated to the RTCA xCELLigence DP workstation (live cell workstation) was prepared, 50 μl of culture medium was added and allowed to equilibrate for 1 h in the incubator, the baseline was measured, 100 µL/well of the prepared cell suspension was added to the CIM plate, and the growth curve was measured.

### In vivo tumor xenograft assay

Briefly, 4-week-old nude mice were randomly divided into two groups (sh-NC and sh-UBE2C groups), with five mice in each group, and the above cancer cells were subcutaneously inoculated (2 × 10^6^ cells/mouse). Tumor formation in mice was observed and recorded every 3 days for 3 weeks, the tumor volume was calculated, and the tumor growth curve was drawn. After 3 weeks, the mice were sacrificed by cervical dislocation, and the xenograft tumors were collected, embedded in paraffin, and stained with HE to observe tumor formation in the xenograft tumors. All animal experiments were approved by the Animal Care Committee of the Air Force Medical University (No. 20220667).

### Statistical analysis

All statistical analysis tools, methods and thresholds used in the article are described in detail in the Materials and Methods section.

## Results

### Identification of cell subtypes in LUAD and differentially expressed genes during the invasive process of LUAD with scRNA-seq

**Fig. 1:**
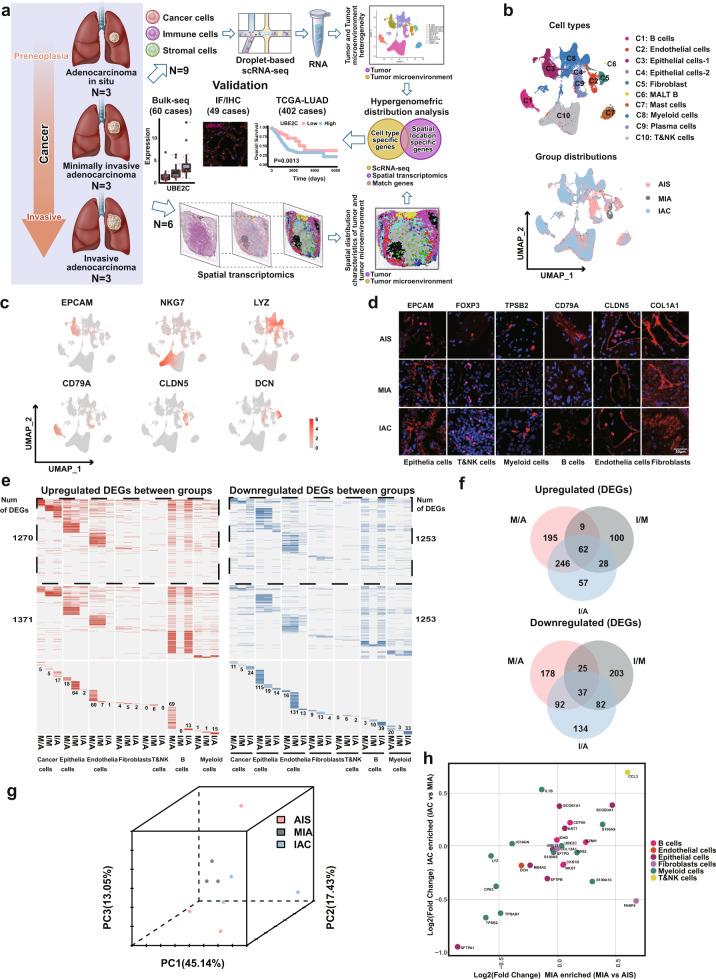
Research design and main findings of this study. **a** The heterogeneity of cancer cells and the TME in early LUAD at three stages (AIS, MIA and IAC) was determined by scRNA-seq. The multicellular spatial environment for each of the three stages was mapped by ST. Hypergeometric distribution analysis was used to integrate cell type-specific and spatial gene expression data obtained by applying scRNA-seq and ST, respectively. Multiple methods were used to validate that the UBE2C cancer cell subcluster and Tregs, NK cells and mast cells in the TME mediate the invasion of early-stage LUAD cells. **b** UMAP showing classification of scRNA-seq data from 115,246 cells from 9 LUAD patients by cell type (left) and pathological stage (right). **c** UMAP of scRNA-seq showing six major cell types identified by marker gene expression (EPCAM: epithelial cells; NKG7: NK/T cells; LYZ: myeloid cells; CD79A: B cells; CLDN5: endothelial cells and DCN: fibroblasts). **d** Protein expression of marker genes determined by IF in six cell types from a TMA consisting of independent LUAD samples (14 AIS cases, 18 MIA cases, 17 IAC cases). **e** Heatmaps of scRNA-seq showing upregulated DEGs (left panel, red) and downregulated DEGs (right panel, blue) by cell type for the following comparisons: MIA versus AIS (M/A), IAC versus MIA (I/M) and IAC versus AIS (I/A). Genes with nonsignificant changes in expression between the two groups are colored and labeled gray. DEGs shared by at least two cell types are shown in the upper portion of the figure (outlined). The middle portion shows DEGs shared by at least two groups, and the lower portion shows DEGs unique to each cell type in each group. The number of genes is marked in the figure. **f** Venn diagrams showing the numbers of common upregulated DEGs (upper panel) and downregulated DEGs (right panel) among the three groups. **g** Principal component analysis (PCA) showing the DEGs among the three groups. The distance between dots represents the difference between groups. **h** Cell type changes in the three stages of LUAD (the abscissa is MIA vs. AIS, and the ordinate is AIC vs. MIA). The *P* value was determined using the Wilcoxon-guided cell ratio test.

To determine the specific cell populations or potential candidate gene signatures involved in the invasion of LUAD, we first collected nine primary LUAD samples, including 3 AIS, 3 MIA and 3 IAC samples (Fig. 1a, Supplementary Fig. 1a, and Supplementary Table 1), for droplet-based scRNA-seq. We obtained a total of 115,246 cells consisting of 37,143 cells (32.2%) from AIS, 30,909 cells (26.8%) from MIA and 47,194 cells (41.0%) from IAC through scRNA-seq, where 25 high-confidence cell clusters were identified based on marker genes (Supplementary Fig. 1b). These cell clusters were broadly divided into ten cell lineages from C1 to C10 (Fig. 1b, left panel), and the principal origin of each cell lineage from AIS, MIA or IAC was further analyzed and depicted (Fig. 1b, right panel). Among them, six major cell types were defined by well-established canonical marker genes^40,41^ (Fig. 1c, Supplementary Fig. 1c, and Supplementary Table 2), including epithelial cells (EPCAM and SCGB1A1), T/NK cells (CD3D, NKG7, GZMK, and GNLY), B cells (CD79A, JCHAIN, and IGHG1), myeloid cells (LYZ, TPSB2, AIF1, HLA-DRA, HLA-DRB1, and CPA3), endothelial cells (CLDN5) and fibroblasts (COL1A1 and DCN). Next, we prepared a TMA containing AIS/MIA/IAC samples and used IF to calibrate the expression of gene markers by the above cells (Fig. 1d and Supplementary Table 3). The percentage of each cell subtype within each cell type (Supplementary Fig. 1d, left panel), as well as the percentage of gene transcript levels of each cell subtype (Supplementary Fig. 1d, right panel) in LUAD tissues, were investigated in depth and summarized.

To further explore the mechanism of the invasive process of LUAD at the cellular level, we compared the gene expression patterns of each cell type, including cancer cells defined by DEGs and CNVs (Supplementary Fig. 1e) as well as six other component cells identified through canonical marker genes (Fig. 1c and Supplementary Fig. 1c), among two of the three groups. During the invasive progression of LUAD, 512 upregulated and 332 downregulated DEGs were identified between the MIA and AIS (M/A) groups, 199 upregulated and 347 downregulated DEGs between the IAC and MIA (I/M) groups, and 393 upregulated and 345 downregulated DEGs between the IAC and AIS (I/A) groups. Notably, approximately half of the upregulated DEGs in the I/A group were already detectable in the M/A group (Fig. 1e, f). PCA of the DEGs revealed that MIA and IAC were very close at the transcriptomic level (Fig. 1g). Further analysis revealed enriched cell types for the three different stages of LUAD (Fig. 1h). To demonstrate the reproducibility of our data, we reclustered cells according to disease stage and patient. As expected, we found the distribution of cell types in three stages and per patient of LUAD to further confirm our results (Supplementary Fig. 2). In addition, to examine individual variations in our scRNA-seq data, we quantified by Spearman correlation and found less individual heterogeneity among the three patients at the same stage (Supplementary Fig. 3). Taken together, these findings suggest that cell-type-specific transcriptional changes contribute to the invasive development of LUAD and that the transition of AIS to MIA is a key process in the early invasion of LUAD.

### The UBE2C+cell subpopulation contributes to the whole invasive process of LUAD

**Fig. 2:**
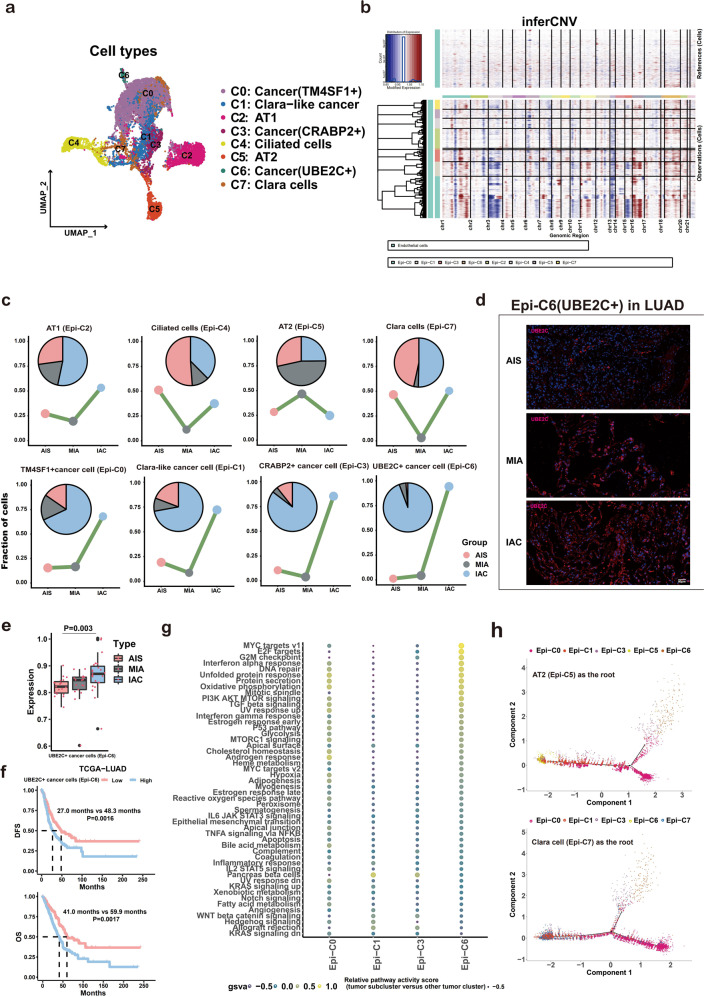
LUAD cells have a polyclonal origin, and the UBE2C + cancer cell subcluster drives the invasion of LUAD. **a** UMAP of scRNA-seq showing eight subclusters consisting of 12,879 epithelial cells from nine LUAD specimens. **b** Large-scale CNVs were observed in four types of cancer subclusters (Epi-C0, Epi-C1, Epi-C3 and Epi-C6) compared to normal endothelial cells. The color shows the log_2_ CNV ratio. Red represents amplifications, and blue represents deletions. **c** Area plot showing changes in eight subtypes of epithelial cells at three pathological stages of LUAD. **d** IF analysis of LUAD TMAs (14 AIS cases, 18 MIA cases and 17 IAC cases) showing cancer cells (Epi-C6: UBE2C + ) in the three stages of LUAD. **e** ssGSEA validated that the expression level of UBE2C + cancer cells increased in the process of cancer invasion from bulk RNA-seq data (20 AIS cases, 17 MIA cases and 23 IAC cases). **f** Survival curves of patients with LUAD according to gene signatures of UBE2C + cancer cells (Epi-C6) in TCGA-LUAD dataset (upper panel: DFS; lower panel: OS). **g** Heatmap of the GSVA results showing differences in pathway activity among different cancer cell subclusters from scRNA-seq data. **h**. Pseudotime analysis by Monocle2 shows the potential evolutionary trajectory of LUAD. Upper: AT2 (Epi-C5) as the root; Down: Clara cell (Epi-C7) as the root.

First, to confirm the pivotal cancer cell subclusters that initiate LUAD invasion, epithelial cells (12,879) were further reclustered into 8 subpopulations (Epi-C0-Epi-C7), including 4 cancer cell subpopulations [Clara-like cancer cells (Epi-C1), TM4SF1 + cancer cells (Epi-C0), CRABP2 + cancer cells (Epi-C3), and UBE2C + cancer cells (Epi-C6)] and 4 normal lung epithelial cell subpopulations [alveolar type I cells (AT1, Epi-C2), alveolar type II cells (AT2, Epi-C5), Clara cells (Epi-C7), and ciliated cells (Epi-C4)] (Fig. 2a, b and Supplementary Fig. 4a) using CNV analysis with endothelial cells as references^9,42–44^. For the cancer cell subtypes, we found that compared with Clara-like cancer cells (Epi-C1) and CRABP2 + cancer cells (Epi-C3), the proportions of both TM4SF1 + (Epi-C0) and UBE2C + (Epi-C6) cancer cells were constantly increased during the invasive process of LUAD and dramatically elevated in IAC. Notably, the proportion change of the UBE2C + subtype was more pronounced than that of the TM4SF1 + subtype from AIS to MIA (Fig. 2c). This suggested that UBE2C + subtype cancer cells may play a more important role in the initiation of LUAD invasion and metastasis, which was further supported by the findings that UBE2C acts as an oncogene and promotes metastasis in other tumors^9,42^. Consistently, UBE2C + subtype cancer cells were found to gradually and stably increase from AIS to IAC using IF and bulk RNA-seq (Fig. 2d, e). Using ssGSEA, we validated the effect of UBE2C + cancer cells (Epi-C6) on the prognosis of patients with LUAD in the TCGA-LUAD database and found that it can be used as a biomarker for predicting the prognosis of LUAD (Fig. 2f). UBE2C + cancer cells (Epi-C6) were mainly enriched in the following gene sets: MYC target V1, cell cycle, AKT and TGF-β signaling pathways and other gene sets; thus, these cells may promote the progression of cancer through these pathways (Fig. 2g). However, neither the expression levels nor the prognostic significance of TM4SF1 was identified using bulk RNA-seq and survival analysis (Supplementary Fig. 4b, c). To investigate the regulatory effects of TFs in different cancer subclusters, we performed SCENIC analysis (Supplementary Fig. 4d). These findings indicate that the accumulation of the UBE2C + cell subpopulation and activation of signaling pathways are implicated in the whole invasive process of LUAD.

To reveal the origin of LUAD at the single-cell level, trajectory analysis was performed to decipher the cell trajectory of LUAD from normal epithelial cells to cancer cells. Both CNV and DEG analysis confirmed the malignant characteristics of cancer cells (Fig. 2b and Supplementary Fig. 4e). Starting from the well-known AT2 cell as the root^45^, the cells were found to first evolve into Clara-like cancer cells (Epi-C1), then into CRABP2 + cancer cells (Epi-C3) and TM4SF1 + cancer cells (Epi-C0), and finally into UBE2C + cancer cells (Epi-C6) as the most poorly differentiated cancer cell type and the end state (Fig. 2h, top panel). Moreover, the pseudotime trajectory axis indicated that Clara cells (Epi-C7) could transdifferentiate into Clara-like cancer cells (Epi-C1) and then two other cancer cell types (Epi-C0 and Epi-C3), while UBE2C + cancer cells (Epi-C6) remained the least differentiated cancer cell type in this track (Fig. 2h, lower panel). IF staining showed the coexistence of Clara-like cancer cells (Epi-C1) and UBE2C + cancer cells (Epi-C6) in LUAD at three different stages (Supplementary Fig. 4f), especially in AIS. Based on these findings, LUAD may originate from both Clara cells and AT2 cells, and the UBE2C + cancer cell subpopulation is the terminal cell type of LUAD.

**Fig. 3:**
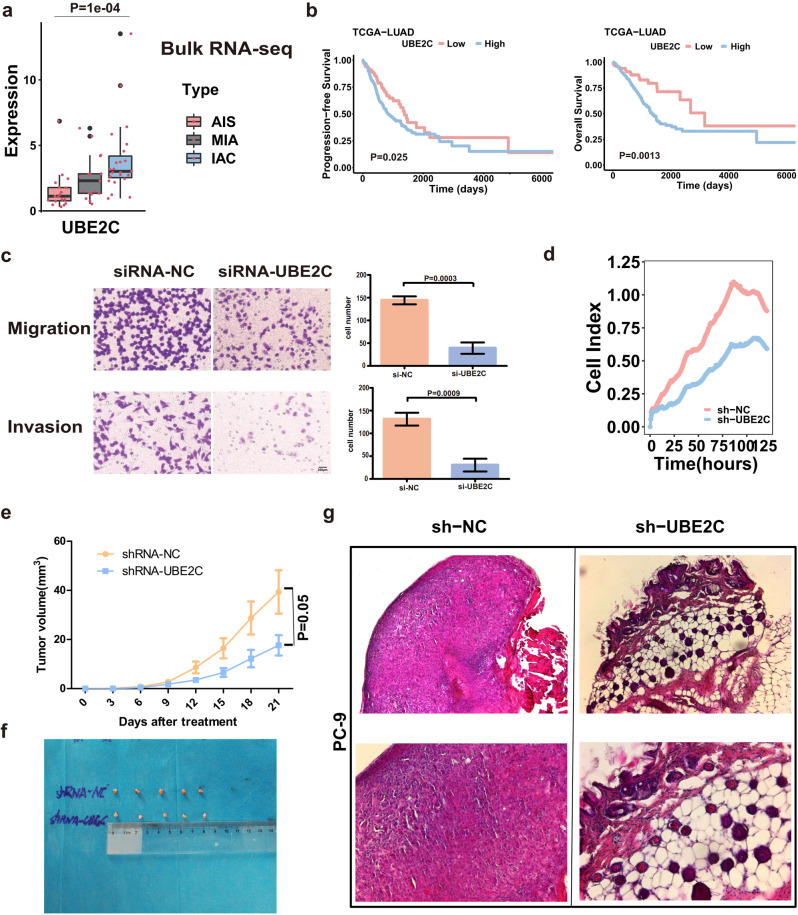
High UBE2C expression promotes the invasion and metastasis of lung adenocarcinoma cells. **a** Bulk RNA-seq analysis (60 cases) validated that the expression level of the UBE2C gene increased in the process of cancer invasion. **b** Kaplan‒Meier analysis (data for 402 LUAD cases from the TCGA database) revealed that patients with high UBE2C expression had worse progression-free survival (upper panel, *P* = 0.025) and OS (lower panel, *P* = 0.0013). **c** A Transwell assay confirmed that knockdown of the UBE2C gene in the PC-9 LUAD cell line decreased cancer cell migration (upper panel) and invasion (lower panel). **d** Cell index assay showing that sh-UBE2C inhibits the proliferation of PC-9 lung cancer cells. **e** Growth curves of xenograft tumors in each group. **f** Subcutaneous tumor size of each group. **g** HE representative images of nude mouse xenografts UBE2C and sh-UBE2C.

As a marker gene of Epi-C6, investigating the UBE2C gene is very important for us to further understand the role of UBE2C + cancer cells (Epi-C6). Using bulk RNA-seq data (60 cases), we verified that UBE2C expression levels gradually increased in the three stages of LUAD (Fig. 3a, Supplementary Table 4). From the TCGA-LUAD dataset (402 LUAD cases), patients with high UBE2C expression had poorer progression-free survival (*P* = 0.025) and overall survival (*P* = 0.0013), as shown in Fig. 3b. Our in vitro experiments confirmed that UBE2C mediates the malignant progression of LUAD by affecting LUAD proliferation and invasion (Fig. 3c, d). Furthermore, in vivo experiments showed that UBE2C silencing significantly attenuated the growth of subcutaneous tumors (Fig. 3e–g). Taken together, these results suggest that UBE2C mediates the proliferation and metastasis of LUAD cells.

### Investigation of distinct TME cell subpopulations in LUAD invasion

**Fig. 4:**
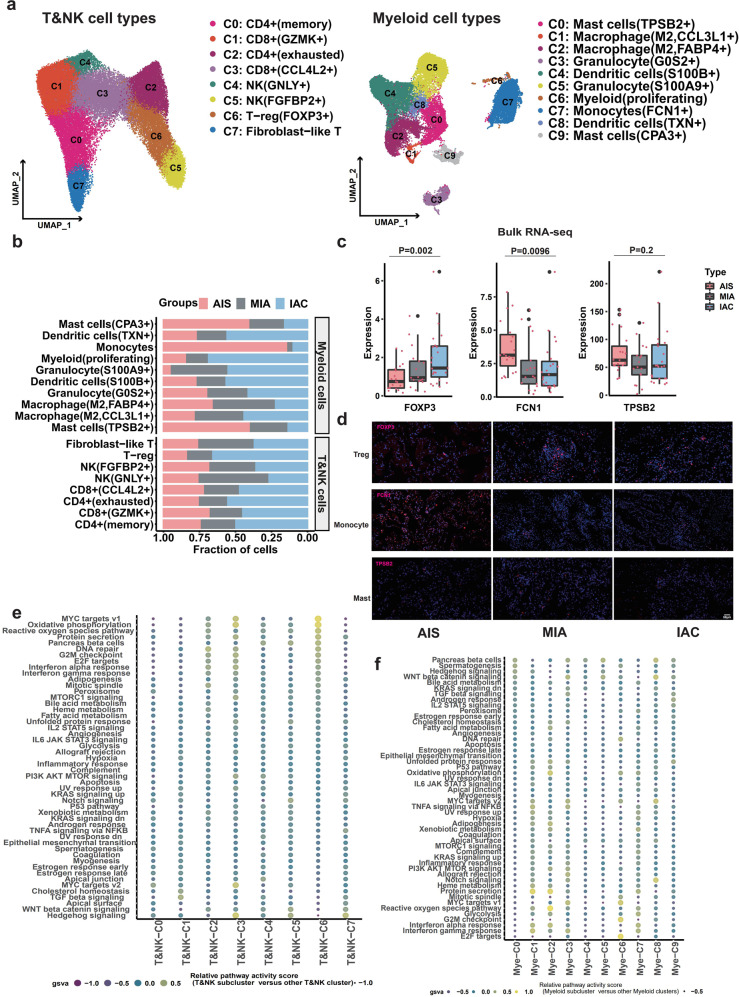
The role of immune cell infiltration in the early-stage LUAD invasion process. **a** UMAP of scRNA-seq showing eight subclusters of T/NK cells (45,612 cells, left) and ten subclusters of myeloid cells (30,627 cells, right). **b** Proportions of eight subclusters of T/NK cells and ten subclusters of myeloid cells in LUAD samples distributed in three stages. **c** Bulk RNA-seq confirmed that the expression of the FOXP3 gene (Tregs, left) gradually increased from AIS to MIA to IAC, while the expression of both the FCN1 gene (monocytes, middle) and the TPSB2 gene (mast cells, right) gradually decreased from AIS to MIA to IAC. **d** IF analysis validated protein expression levels during the LUAD invasion process in TMAs. The protein expression levels of the FCN1 gene (monocytes, middle panel) and TPSB2 gene (mast cells, lower panel) decreased, while those of the FOXP3 gene (Tregs, upper panel) increased from AIS to MIA to IAC. **e** Heatmap of GSVA results showing differences in pathway activity among different T/NK-cell subclusters (left panel) from scRNA-seq. **f** Heatmap of GSVA results showing differences in pathway activity among different myeloid cell subclusters from scRNA-seq.

To explore the changes in different TME cell types during the invasive process of LUAD, each cell type of the five major TME cell populations (Fig. 1c) was further investigated. First, T/NK cells and myeloid cells were subclassified by clustering analysis. As shown in Fig. 4a, 8 subtypes of T/NK cells, including 2 subtypes of CD4 + T cells (T/NK-C0: LTB + and T/NK-C2: CXCL13 + ), 2 subtypes of CD8 + T cells (T/NK-C1: GZMK + and T/NK-C3: CCL4L2 + ), 2 subtypes of NK cells (T/NK-C4: GNLY + and T/NK-C5: NKG7 + ), 1 subtype of Tregs (T/NK-C6: FOXP3 + ) and 1 subtype of fibroblast-like T cells (T/NK-C7: HSPA1B + ) (Fig. 4a, left panel), and 10 subtypes of myeloid cells, including 2 subtypes of mast cells (Mye-C0: TPSB2 + and Mye-C9: CPA3 + ), 2 subtypes of macrophages (Mye-C1: CCL3 L and Mye-C2: FABP4, anti-inflammatory), 2 subtypes of dendritic cells (Mye-C4: S100B + and Mye-C8: TXN + ), 2 subtypes of granulocytes (Mye-C3: GOS2 + and Mye-C5: S100A9 + ), one subtype of monocytes (Mye-C7: FCN1 + ) and one subtype of myeloid cells (Mye-C6, proliferating) (Fig. 4a, right panel) were identified. Among them, the percentages of mast cells (Mye-C0: TPSB2 + ) and monocytes (Mye-C7: FCN1 + ) gradually and consistently decreased from AIS and MIA to IAC (Fig. 4b). However, the percentage of NK cells (T/NK-C4: GNLY + ) was only increased from AIS to MIA, while the abundance of Tregs (T/NK-C6: FOXP3 + ) was dramatically elevated from MIA to IAC (Fig. 4b). Consistently, bulk RNA-seq and IF assays further confirmed the patterns of cell subpopulation alterations (Fig. 4c, d). The potential hallmark signaling pathways implicated in these cell subpopulation alterations were further analyzed using GSVA. As shown in Fig. 4e, f, cell cycle- and cell growth- or proliferation-related pathways, including PI3K/AKT/MTOR signaling, G2M checkpoint, E2F targets, and MYC targets V1, were highly enriched in NK (T/NK-C4: GNLY + ) and Tregs (T/NK-C6: FOXP3 + ); Hedgehog, WNT β-catenin and interferon signaling were pronounced in mast cells (Mye-C0: TPSB2 + ) and monocytes (Mye-C7: FCN1 + ). Therefore, we proposed that activation of tumor-associated pathways mediated by an increase in Tregs and a decrease in monocytes promotes early LUAD invasion.

Similarly, among 8 B-cell subtypes, 5 endothelial subtypes and 7 fibroblast subtypes (Supplementary Fig. 5a and 6a), we found that the fraction of MALT B cells (C4: IGHD + ) was markedly reduced from AIS to MIA/IAC, and the fraction of secretory B cells (C7: GZMB + ) was dramatically increased from MIA to IAC (Supplementary Fig. 5b). Furthermore, the proportion of lymphatic endothelial cells (End-C4: PDPN + ) was constantly decreased from AIS to IAC (Supplementary Fig. 6b), while the fraction of cancer-associated fibroblasts (C1) was significantly decreased in IAC compared with AIS and MIA (Supplementary Fig. 6b). Bulk RNA-seq and IF assays further demonstrated similar expression patterns of corresponding cell type markers from AIS to IAC (Supplementary Fig. 5c, d and Supplementary Fig. 6c, d). GSVA revealed that oxidative phosphorylation, DNA repair, and reactive oxygen species pathways were highly enriched in secretory B cells-C7 and TGF-β signaling in MALT B cells-C4 (Supplementary Fig. 5e, f); Notch signaling, oxidative phosphorylation, MYC target V2, reactive oxygen species pathway, and TGF-β signaling were pronounced in lymphatic endothelial cells (End-C4: PDPN + ), and oxidative phosphorylation, MYC signaling, DNA repair, WNT β-catenin signaling, TGF-β signaling, and epithelial mesenchymal transition (EMT) were pronounced in cancer-associated fibroblasts (C1) (Supplementary Fig. 6e, f). Notably, these results suggest that TGF-β signaling activation in TMEs is involved in the invasion process, which is consistent with previous studies^46,47^. Collectively, these results suggest that a constant decrease in mast cells (Mye-C0), monocytes (Mye-C7) and lymphatic endothelial cells (End-C4) may be implicated in the whole invasive process of LUAD. In the early stage of invasive LUAD, the increases in NK cells (T/NK-C4) and MALT B cells (B-C4) were more pronounced from AIS to MIA; conversely, the increases in Tregs (T/NK-C6) and secretory B cells (B-C7) were identified in the late stage of invasive LUAD (MIA to IAC). To verify the impact of individual differences on our conclusions, we quantified the reproducibility of subclusters of each major cell type by the Wilcoxon test (Supplementary Fig. 7) and IDR analysis (Supplementary Table 5).

### The reciprocal interaction between cancer cells and TME cells induces activation of TGF-β signaling in IAC

**Fig. 5:**
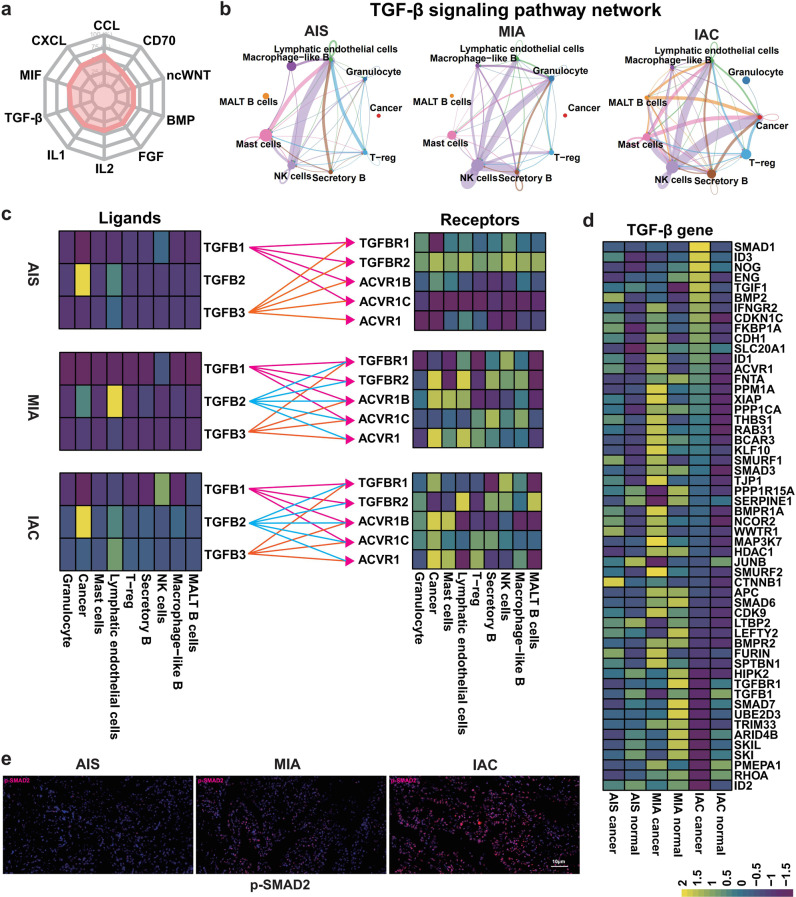
Maps showing the role of the TGF-β signaling pathway in the dialog between cancer cells and the TME. **a** Radar chart from CellChat data showing the major signaling pathways that mediate cell-to-cell interactions in LUAD. **b** TGF-β signaling pathway interactions between cancer cells and nine specific cell types of the TME from scRNA-seq data. The maps show TGF-β pathway interactions between cancer cells and cells of the immune microenvironment in AIS (left panel), MIA (middle panel), and IAC (right panel). **c** Heatmaps showing the distributions of ligands and receptors in the TGF-β pathway in the three stages of LUAD (upper: AIS, middle: MIA, lower: IAC). **d** GSVA of scRNA-seq showing that differences in the expression of genes (54 genes) downstream of the TGF-β pathway between normal cells and cancer cells differed among the three stages of LUAD. **e** TMAs were used to verify the expression of a gene related to TGF-β phosphorylation (p-Smad2) in the three stages of LUAD.

Cell signaling and communication between cancer cells and the TME are crucial for the progression and metastasis of cancer^48,49^. CellChat analysis revealed the top ten interacting pathways among cells in LUAD based on our scRNA-seq results (Fig. 5a). Among these pathways, the TGF-β pathway was further selected for downstream analysis because of its pivotal role in cancer invasion and metastasis^50,51^. Surprisingly, no communication via TGF-β signaling between cancer cells and TME cells was observed within the AIS and MIA, but this interaction was significantly strengthened in IAC, especially in NK, mast and MALT B cells (Fig. 5b), which is consistent with the change in the number of cells of interest in the previous single-cell data results. Notably, this differential interaction of TGF-β signaling between cancer cells and TME cells in the different stages of LUAD may be largely attributed to the differential expression of TGF-β ligands or receptors in different cell types. In AIS and MIA, cancer cells and TME cells expressed low levels of TGF-β ligands (Fig. 5c and Supplementary Table 6). It is conceivable that communication via TGF-β signaling between cancer cells and TME cells was relatively scant due to the lack of abundant stimulation of TGF-β ligands. However, in the IAC, the levels of TGF-β ligands were widely upregulated in both cancer cells and TME cells (Fig. 5c), which suggested that both autocrine and paracrine mechanisms may simultaneously promote constitutive communication of TGF-β signaling between cancer cells and TME cells in IAC. This differential interaction may be associated with the complex and sometimes paradoxical role of TGF-β signaling in cancer: in early stages, it inhibits cell growth as a tumor-suppressive pathway, while in later stages, TGF-β promotes invasion and metastasis^52^. Consistently, the expression levels of downstream targeted genes, as well as the downstream factor p-SMAD2 of the TGF-β pathway, were highly activated in IAC compared with AIS and MIA (Fig. 5d, e, and Supplementary Table 7). Therefore, these results imply that communication and interaction of TGF-β signaling between cancer cells and TME cell-induced unrestrained activation of TGF-β signaling in IAC may play a key role in the invasion of late-stage LUAD.

Next, we used NicheNet to explore cell-to-cell communication during the three stages of LUAD. As shown in Supplementary Fig. 8, we concluded that TGF-β pathway-related genes were mainly expressed in cancer and NK cells, which is consistent with the prediction results of CellChat. Moreover, ligand and receptor prediction revealed that TGF-β pathway communication is mainly achieved through the expression of ACVRL1, TGFBR1, and TGFBR2 in LUAD. Consistently, the expression levels of downstream target genes and downstream genes of the TGF-β pathway, including SMAD3, SMAD 6, and SMAD 7, were highly activated in the three stages of LUAD (Supplementary Fig. 9).

### Spatial distribution of Tregs in cancer regions mediates LUAD invasion

**Fig. 6:**
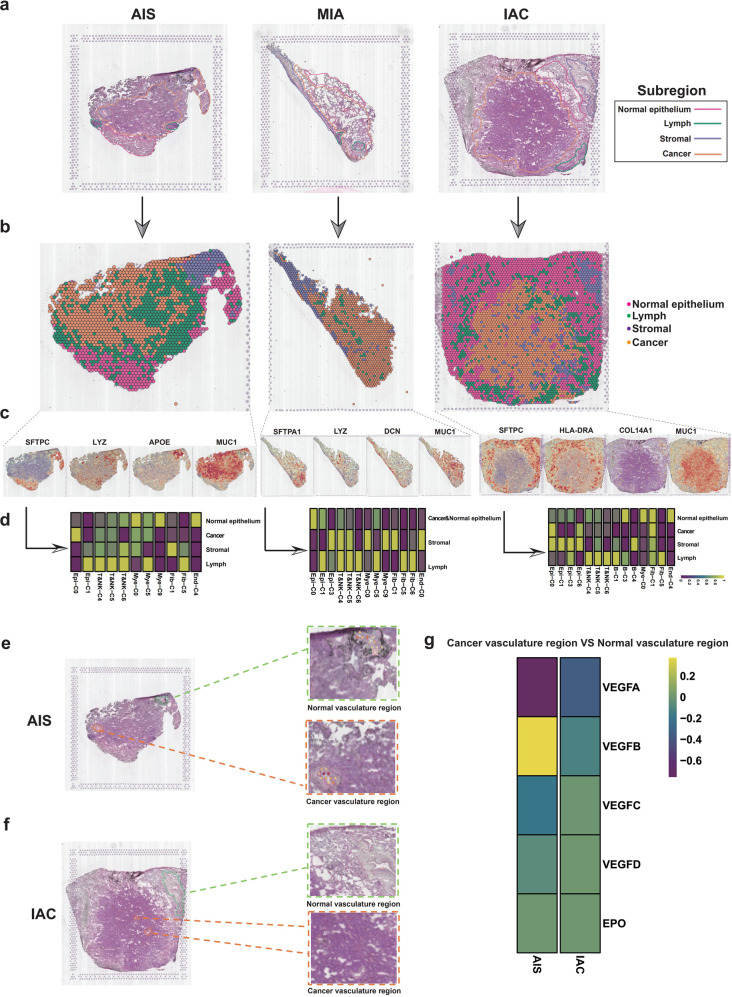
Effect of the spatial distribution of the TME on the invasion of early-stage LUAD. **a** HE staining showing histologically distinct regions of AIS (TD8), MIA (TD3) and IAC (TD1) samples. Pink: normal epithelium region; green: lymph region; purple: stromal region; yellow: cancer region. **b** Spatial transcriptome atlas depicting the spatial regions (cancer region, stromal region, lymph region and normal epithelial region) of samples representing the three stages of LUAD. **c** Marker genes of the four spatial regions: cancer region, stromal region, lymph region and normal epithelial region. **d** Heatmaps obtained upon hypergeometric distribution analysis of all specific cell types identified by scRNA-seq analysis and regions defined by ST analysis (left panel: AIS, TD8; middle panel: MIA, TD3; right panel: IAC, TD1). **e** Identification of the cancer vasculature region and normal vasculature regions in AIS patient (TD8) by ST analysis. **f** Identification of the cancer vasculature region and normal vasculature regions in IAC patient (TD1) by ST analysis. **g** Expression of vascular endothelial cell-related (angiogenesis-related) genes in different vascular regions.

Related studies have suggested that the spatial distribution of TME cells and their density changes are equally important in the malignant characterization of cancers^53,54^. To map the spatial architecture of LUAD, six specimens (AIS: TD5, TD8; MIA: TD3, TD6; and IAC: TD1, TD2) analyzed by scRNA-seq were further subjected to ST. Based on HE staining, we annotated the different morphological regions of the slices and divided the samples into four different histological regions, including the cancer region, normal epithelial region, stromal region, and lymph region (Fig. 6a). In addition, to examine individual variations in our ST data, we quantified the spots of each region by Spearman correlation (Supplementary Fig. 10) and IDR analysis (Supplementary Table 8). To integrate the scRNA-seq information into spatial architecture, a hypergeometric distribution analysis was carried out.

AIS specimen TD8 was divided into four regions by ST (Fig. 6b), and the expression of marker genes further confirmed the accuracy of the spatial division (Fig. 6c). As expected, TM4SF1 + cancer cells (Epi-C0), 2 subtypes of NK cells (T/NK-C4: GNLY and T/NK-C5: NKG7) and mast cells (Mye-C0: TPSB2) were colocalized in the cancer region, while Tregs (T/NK-C6: FOXP3) were not found in the cancer region (Fig. 6d). Similarly, another AIS sample TD5 was divided into the same four final histological regions by ST (Supplementary Fig. 11a, b). The hypergeometric distribution showed that in the cancer region, TM4SF1 + cancer cells (Epi-C0) colocalized with the two subtypes of NK cells (T/NK-C4: GNLY and T/NK-C5: NKG7) and mast cells (Mye-C0: TPSB2, Mye-C9: CPA3) (Supplementary Fig. 11c). GSVA revealed that the TGF-β signaling pathway was enriched in the cancer region identified by ST (Supplementary Fig. 11d). These data support the notion that immune surveillance in the AIS stage is well functioning and can prevent Tregs from infiltrating the cancer region.

The MIA sample (TD3) was also divided into four distinct regions by ST (Fig. 6b). Hypergeometric distribution analysis showed that TM4SF1 + cancer cells (Epi-C0) and Clara-like cancer cells (Epi-C1) were highly enriched in the cancer region, and NK cells (T/NK-C4: GNLY and T/NK-C5: NKG7) and mast cells (Mye-C0: TPSB2) colocalized with the above two cancer cell subclusters, while Tregs (T/NK-C6: FOXP3) and mast cells (Mye-C9: CPA3) were absent from the cancer region (Fig. 6d). In another case of MIA (TD6), the cancer cell subtypes enriched in the cancer region, including Clara-like cancer cells (Epi-C1), NK cells (T/NK-C4: GNLY and T/NK-C5: NKG7), Tregs (T/NK-C6: FOXP3) and mast cells (Mye-C0: TPSB2, Mye-C9: CPA3), were also located in the cancer region (Supplementary Fig. 11c). In addition, the cancer region of TD3 was enriched in oxidative phosphorylation, DNA repair, E2F targets, and TNFα/NF-kB signaling (Supplementary Fig. 11d).

Finally, spatial and cellular data corresponding to two cases of IAC were further investigated. In sample TD1, the cancer region, normal epithelial region, stromal region and lymph region were clearly distinguished by ST (Fig. 6b), which was highly consistent with the HE staining results (Fig. 6a). Hypergeometric distribution analysis showed that TM4SF1 + cancer cells (Epi-C0) and UBE2C + cancer cells (Epi-C6) were enriched in the cancer region, and CAFs (Fib-1) and Tregs (T/NK-C6: FOXP3) colocalized with the above cancer cell subclusters in the cancer region (Fig. 6d). Similarly, TD2 was also shown to contain a cancer region by HE staining analysis but had no clear stromal region or lymph region (Supplementary Fig. 11a, b). The cancer cell subtypes enriched in the cancer region were CRABP2 + cancer cells (Epi-C3), and NK cells (T/NK-C5: NKG7) and mast cells (Mye-C0: TPSB2, Mye-C9: CPA3) were also located in the cancer region (Supplementary Fig. 11c). GSVA analysis showed that the cancer region of TD2 (IAC) was enriched in KRAS signaling down (Supplementary Fig. 11d). These data demonstrate the extensive infiltration of Tregs into the cancer region as LUAD progresses to the IAC stage.

Interestingly, by comparing the vasculature region in cancer and the normal region in AIS and IAC samples, we found that the cancer vasculature region of IAC presented a more pronounced number of blood vessels in H&E (Fig. 6e, f) and higher expression levels of VEGFs (Fig. 6g) compared with the normal vasculature region. Conversely, both the density of blood vessels and the expression levels of VEGFs between normal and cancer vasculature regions of AIS did not produce obvious differences (Fig. 6e–g). This finding suggested that angiogenesis may significantly contribute to the invasive process of LUAD.

To categorize the mixed cell types of each spot, we performed the RCTD method on spatially resolved transcriptomic data with the paired scRNA-seq data. Clara-like cancer cells, as a less malignant cancer cell type of early-stage LUAD, were identified in both specimens of AIS (Supplementary Fig. 12 and Supplementary Fig. 13), which is consistent with our single-cell results as well as the cancer cell pathological features of AIS of the lung. When LUAD progressed to the IAC stage, RCTD data showed that UBE2C + cancer cells infiltrated the cancer region (Supplementary Fig. 12). To map the per-cell gene signatures from single-cell sequencing data to each spatial spot, we performed Cell-ID, which is a clustering-free multivariate statistical method (Supplementary Fig. 14). Interestingly, TM4SF1 + cancer cells were found in AIS, MIA and IAC. Further analysis showed that in AIS, except for TM4SF1 + cancer cells, the remaining cancer cells were mainly Clara-like cancer cells, and the biological behavior of such cells was relatively mild. However, in IAC, the most malignant UBE2C + cancer cells gradually dominate, which may explain why the prognosis of IAC is poor. The above results are basically consistent with the results of hypergeometric distribution analysis, but this method is not satisfactory for the identification of the spatial distribution of TME cells.

Taken together, our results indicate that as LUAD progresses from precancer to IAC, Tregs are spatially recruited in cancer regions, while NK-cell and mast cell infiltration are absent from this region. Moreover, we found that abnormalities in cancer blood vessels play an important role in the invasion of LUAD from AIS to IAC.

### The peripheral cancer region was more active than the central cancer region analyzed by ST

**Fig. 7:**
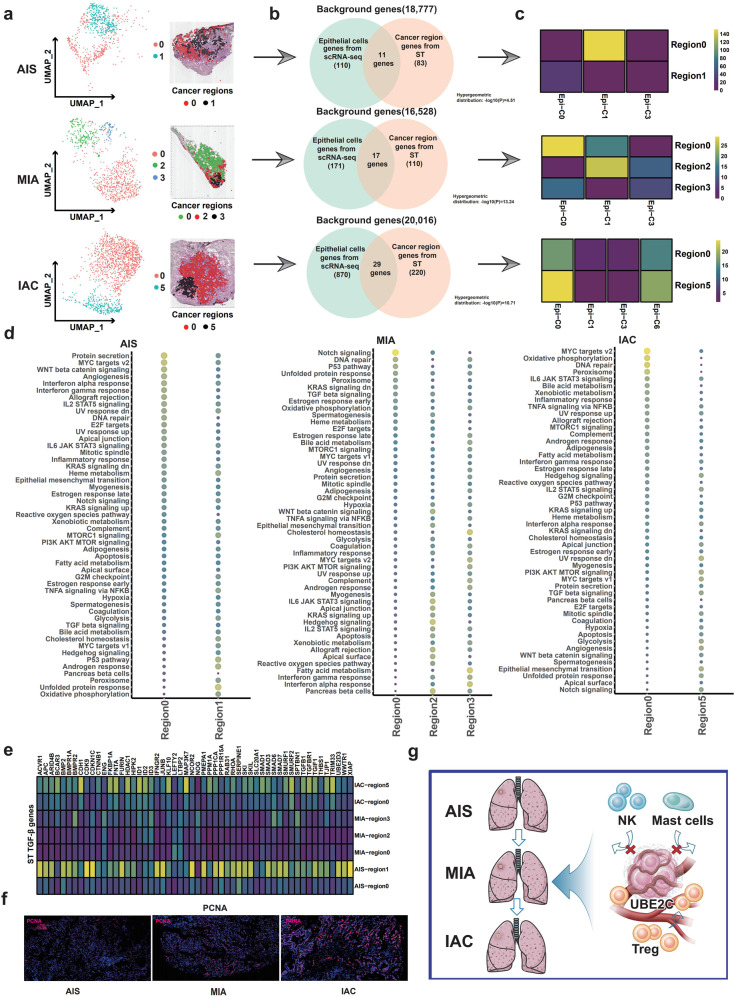
Spatial heterogeneity of cancer cells. **a** ST analysis was used to subregionalize the cancer region in the HE-stained cancer region (upper panel: AIS, TD8; middle panel: MIA, TD3; lower panel: IAC, TD1). **b** Venn diagram showing overlapping genes specific for cell type of scRNA-seq data and tissue region of ST data from among 33,538 genes. **c** The hypergeometric distribution method was applied to match the four cancer subtypes identified by scRNA-seq data with the cancer location-specific genes identified by ST data. **d** Heatmap of GSVA results showing differences in the pathway activity in different spatial cancer subregions (left panel: AIS, TD8; middle panel: MIA, TD3; right panel: IAC, TD1). **e** GSVA data showing differences in the expression of genes downstream of the TGF-β pathway in cancer cells with different spatial distributions. **f** IF validated differences in the activity of cancer cells with different spatial distributions (PCNA expression). **g** Hypothetical models illustrating that the UBE2C + cancer cell subcluster increases and recruits Tregs, repels NK cells and mast cells, and promotes the invasive progression of LUAD from AIS to IAC.

To track the spatial distribution and biological significance of the four cancer subpopulations identified by scRNA-seq, we reclustered the cancer region into subregions by ST. In the AIS sample of TD8, the cancer-rich regions in the slices were further divided into transcription-related subregions, which consisted of a central region (region 1, black) and the peripheral region (region 0, red) (Fig. 7a). The hypergeometric distribution was used to identify overlapping cell type-specific scRNA-seq gene expression data and spatial ST gene expression data (Fig. 7b). We found that Clara-like cancer cells (Epi-C1) were highly enriched in the peripheral region (region 0), and the central region (region 1) was dominated by TM4SF1 + cancer cells (Epi-C0) (Fig. 7c). GSVA showed that several oncogenic signaling pathways, including Wnt/β-catenin signaling, MYC target v2 and angiogenesis pathways, were highly activated in the peripheral region (region 0), which were not observed in the central region (region 1) (Fig. 7d, left panel). This finding suggested that the peripheral cancer region may display more active oncogenic features than the central cancer region, supporting the notion that the acquisition of invasive properties of cancer cells may be more likely to originate from the peripheral region than from the central region. Notably, TGF-β signaling pathway-related genes were highly expressed in the central region compared to the peripheral region (Fig. 7e). Given the finding that TGF-β signaling inhibits cell growth in the early stages of cancer^52^, the central region of AIS may seem to be inert compared with the active region (peripheral region). Consistently, proliferation indices, such as PCNA, were highly expressed in the peripheral region compared with the central region (Fig. 7f). We also used monocle 3 implemented in SPAPA to perform a pseudotime analysis of different regions (edge and core) of the tumor, which confirmed our results, showing a trend of differentiation from the center to the edge (Supplementary Fig. 15).

In MIA the cancer region of the slice from sample TD3 was partitioned into three subregions: cancer regions 0, 2 and 3 (Fig. 7a). Consistent with AIS, central cancer subregion 0 of TD3 was mainly dominated by TM4SF1 + cancer cells (Epi-C0), while peripheral cancer region 2 was mainly dominated by Clara-like cancer cells (Epi-C1) (Fig. 7c). However, the potential pathways enriched in the peripheral and central regions of MIA varied from those in AIS. As shown in Fig. 7d (middle panel), the EMT pathway was highly activated in peripheral region 2 but inert in central region 0. The MIA sample TD6 had two more subregions (regions 5 and 6) (Supplementary Fig. 16a, b). Clara-like cancer cells (Epi-C1) were highly enriched in region 6, and TM4SF1 + cancer cells (Epi-C0) were highly enriched in regions 4 and 5 (Supplementary Fig. 16c). GSVA showed that region 0 mainly showed activation of PI3K/AKT signaling that was inhibited in the remaining cancer regions (Supplementary Fig. 16d).

Finally, by analyzing IAC samples (TD1, TD2), we found that the cancer region could be separated into two cancer subregions 0 and 5 with clear boundaries (Fig. 7a). Both cancer regions 0 and 5 were highly enriched with TM4SF1 + cancer cells (Epi-C0) and UBE2C + cancer cells (Epi-C6), especially region 5 (Fig. 7c), which was consistent with the findings of scRNA-seq that the proportions of both TM4SF1 + (Epi-C0) and UBE2C + (Epi-C6) cancer cells were dramatically elevated in IAC (Fig. 2c). The DNA repair, MYC target v2, and oxidative phosphorylation pathways were highly activated in cancer region 0 compared with those in cancer region 5 (Fig. 7d). TGF-β signaling pathway-related genes were highly expressed in cancer region 5 compared with region 0 (Fig. 7e). In addition, sample TD2 was divided into four subregions (Supplementary Fig. 16a, b). Regions 0 and 5 of the cancer overlapped with TM4SF1 + cancer cells (Epi-C0), and regions 1 and 3 of the tumor overlapped mainly with CRABP2 + cancer cells (Epi-C3) (Supplementary Fig. 16c). The cancer-related pathways (EMT, MYC target v2, and oxidative phosphorylation pathways) were mainly activated in region 3 of the tumor, located in the peripheral area (Supplementary Fig. 16d). Collectively, as LUAD progresses from AIS to IAC, cancer cells show increasingly clear spatial distribution, the malignant features of tumor margins are more prominent, and the spatial distribution of cancer cells may be more important than the type of cancer cells.

In conclusion, these findings indicate that the process of AIS to MIA is a confirmed key step for LUAD invasion, and the UBE2C + cancer cell subpopulation was found to play a vital role in driving this process. Multiomics spatial mapping of LUAD proved that TGF-β signaling interactions between cancer cells and the TME and spatial changes that regulate immune escape are involved in LUAD invasion. (Fig. 7g).

## Discussion

Due to the limitations of traditional bulk RNA-seq, our current understanding of the dynamics of LUAD invasion from AIS to IAC is rudimentary. Here, we identified four cancer cell subpopulations at the single-cell level. The UBE2C + cancer cell subpopulation was involved in the early and late stages of the LUAD invasion process, suggesting that UBE2C may be the initiator in LUAD invasion, especially in the scenario that UBE2C is found to be a driver gene in the metastasis of LUAD and other tumors^55–58^. The spatial distribution of these four cancer cell subpopulations of interest was also the focus of our research. To the best of our knowledge, we mapped the spatial heterogeneity of LUAD for the first time to integrate cell type-specific scRNA-seq data and position-specific ST data through hypergeometric distribution analysis^16,59^. Interestingly, UBE2C + cancer cells are mainly distributed in the IAC and in the peripheral cancer region, which represent more active tumor biological behavior^60,61^. In light of these findings, UBE2C may serve as a candidate gene for the pathological identification of preneoplasia and IAC of LUAD.

Controversy regarding the cellular origin of LUAD has seized great momentum recently. The traditional point of view states that LUAD mainly originates from AT2 cells^45,62^. However, several independent studies have reported that Clara cells may be the cellular origin of LUAD^63,64^. Sutherland et al.^65^ proved that both KRAS activation and TP53 loss contributed to the transformation of Clara cells and AT2 cells into malignant LUAD cells. However, Trp53F/F mice infected with Adeno5–SPC–Cre and Adeno5–CC10-Cre viruses exhibit differences in tumor profiles, indicating that LUAD originates from both AT2 and Clara cells by varying mechanisms. Even from the same origin, activation of KRAS induced hyperplasia and adenoma from Clara cells during embryonic development but only led to pulmonary hyperplasia in adults^66^, suggesting that oncogenic transformation of Clara cells depends on the developing stage. Pseudochronological analysis of scRNA-seq data to predict the differentiation potential of cells has been used to study the origin of tumor cells^67,68^. In this study, we demonstrated that Clara-like cancer cells (Epi-C1) coexisted with three other LUAD subtypes in three stages of LUAD, and pseudochronological analysis of Clara cells and AT2 cells demonstrated that both can be converted to LUAD cells. These data at the single-cell level in combination with other independent studies at the genetic level provided evidence that LUAD could originate from both Clara cells and AT2 cells. In this study, we demonstrated that Clara-like cancer cells (Epi-C1) coexisted with three other LUAD subtypes in three stages of LUAD, and pseudochronological analysis of Clara cells and AT2 cells demonstrated that both can be converted to LUAD cells. These data at the single-cell level in combination with other independent studies at the genetic level provided evidence that LUAD could originate from both Clara cells and AT2 cells.

Changes in the TME during cancer evolution from AIS to MIA to IAC suggest that the early carcinogenesis of LUAD is a progressive process formed by host immune surveillance^4,9,10^. In a recent study, NK and MALT B cells were demonstrated to be obviously increased in the early stage of invasive LUAD (AIS to MIA) compared with the increase in Treg and secretory B cells in the late stage of invasive LUAD (MIA to IAC). These results were consistent with previous research^9,10^. In addition, mast cells and monocytes were identified to be constantly decreased during the invasive process of LUAD, suggesting that myeloid cells were also involved in the invasion of LUAD. Notably, from the ST results, in AIS, there was no Treg infiltration in the cancer region, while in IAC, cancer cells recruited Tregs into the cancer region, suggesting that the accumulation of Tregs in the cancer region initiates the early invasion process of LUAD. Interestingly, the strong interaction-induced activation of TGF-β signaling between cancer cells and the TME was identified in IAC, which was not observed in AIS and MIA, consistent with the crucial and established role of TGF-β signaling in the late stage of cancer metastasis^52,69^. Therefore, an in-depth understanding of the DEGs and specific cell subpopulations during the separate stages of invasive LUAD, as well as the interaction and communication between cancer cells and the TME in the invasive process of LUAD, will facilitate the early detection of metastatic LUAD and the development of targeted therapy against LUAD at different stages.

Limitations of our study should be noted when interpreting and extrapolating our data. First, individualized progression of invasive LUAD could not be neglected and assessed in separate patients. Second, tumor heterogeneity should be analyzed in detail in more sections of the same tissue, although the tumor tissues and slices were optically maintained. Last but not least, since only individual spatial information was analyzed separately, it was not possible to integrate comprehensive spatial information for all LUAD patients due to the limitations of ST techniques for comparative analysis between groups.

In summary, we mapped a genetic and spatial atlas of the dynamic evolution of invasive LUAD using a multiomics approach consisting of synchronous scRNA-seq and ST to determine the DEGs, specific cell subpopulations and cell interactions, which will be helpful and beneficial for the identification and development of individualized therapeutic strategies in invasive LUAD.

## Supplementary information

Supplementary tables and figures

## Data Availability

All the original data generated in this study are included in this article and its supplementary files. The raw sequencing data and processed gene expression data of single-cell RNA sequencing and spatial transcriptomics were deposited at Gene Expression Omnibus (GEO) under the accession codes GSE189357 and GSE189487, respectively.
