# Supplementary material for: Delineating the dynamic evolution from preneoplasia to invasive lung adenocarcinoma by integrating single-cell RNA sequencing and spatial transcriptomics
Source: Exp Mol Med. 2022 Nov 25;54(11):2060–76. doi: 10.1038/s12276-022-00896-9 (PMC9722784; doi:10.1038/s12276-022-00896-9)
Supplement: Supplementary file 1 — Supplementary tables and figures [file 12276_2022_896_MOESM1_ESM.pdf]

Supplementary tables

Supplementary table 1. Clinical characteristics of patients with LUAD from whom scRNA-seq and ST data were obtained.

No.	Gender	Age	Smoking status	Tumor location	Tumor diameter	Radiological type	Histological type
TD1	Female	57	Never	Left upper lobe	17mm	SN	IAC
TD2	Male	47	Never	Right lower lobe	20mm	SN	IAC
TD3	Male	37	Never	Left upper lobe	10mm	pGGN	MIA
TD4	Female	45	Never	Left upper lobe	12mm	pGGN	MIA
TD5	Female	56	Never	Left upper lobe	16mm	SSN	AIS
TD6	Female	56	Never	Right upper lobe	21mm	SSN	MIA
TD7	Female	40	Never	Left lower lobe	8mm	SSN	AIS
TD8	Male	69	Ever	Left upper lobe	7mm	pGGN	AIS
TD9	Female	62	Never	Right upper lobe	15mm	SSN	IAC

Supplementary table 2. List of marker genes for major six types of cells.

Cell type	Epithelial cells	T/NK cells	Myeloid cells	B cells	Endothelial cells	Fibroblasts
Marker genes	CAPS	GZMK	LYZ	JCHAIN	CLDN5	DCN
	SCGB1A1	GZMB	CTSD	IGHM	RAMP2	LUM
	WFDC2	NKG7	CCL18	IGHG1	CAV1	COL1A2
	KRT8	GNLY	CD68	IGHA1	VWF	COL3A1
	KRT19	KLRD1	CD14	IGHG4	PECAM1	COL1A1
	SCGB3A1	KLRC1	HLA-DRA	IGHA2	CDH5	FN1
	SCGB3A2	TIGIT	HLA-DRB1	IGHG3	EMCN	COL6A2
	KRT18	LTB	TNF	IGHG2	CD34	COL6A3
	EPCAM	TNFRSF4	FCGR3A	MZB1	CD31	ACTA2
	MUC1	TNFRSF18	CD63	IGHD	THBD	COL6A1
	SFTPC	IL2RA	CD163	CD79A		COL5A2
	SFTPA1	GZMH	FCGR2A	MS4A1		COL4A2
	SFTPA2	CD3D	TPSB2			
	SFTPB	FOXP3	TPSAB1			
	PGC		CPA3			
	AGER		HPGDS			
	CAV1		MS4A2			
	CYP4B1		AIF1			

	KRT7	IL1RN
	ID1	CD86
	SFTPD	THBD
	CDH1	S100A9
		S100A8
		CXCL8
		FCGR3B
		IL1RN
		PTGS2
		CD1E
		FCGR2A
		THBD
		HLA-DPB1
		CD1C
		CD40

Supplementary table 3. Clinicopathological characteristics of patients with early LUAD verified by IF analysis.

Variable			Histological type			
	n	AIS(14)	n	MIA(18)	n	IAC(17)	P value
Gender							0.573
Male	5		7		8		
Female	9		11		9		
Age							0.794
⩾65	2		4		4		
＜65	12		14		13		
Smoking status							0.100
Never	12		14		9		
Ever	2		4		8		
Family history of tumor							0.178
Absent	13		13		11		
Present	1		5		6		
Tumor diameter							0.092
0-10mm	5		1		1		
10-20mm	6		13		11		
20-30mm	3		3		5		
Tumor location							0.781
RUL	5		6		6		
RML	1		1		0		

RLL	2	4	3	
LUL	4	5	8	
LLL	2	2	0	
Radiological type				< 0.001
pGGN	7	4	1	
SSN	6	14	8	
SN	1	0	8	
Surgical approach				0.005
Lobectomy	7	8	16	
Sub-lobectomy	7	10	1	

Supplementary table 4. Clinicopathological characteristics of patients with early LUAD verified by bulk RNA-seq.

Variable			Histological type				
	n	AIS(20)	n	MIA(17)	n	IAC(23)	P value
Gender							0.377
Male	4		6		9		
Female	16		11		14		
Age							0.323
⩾65	3		4		8		
< 65	17		13		15		
Smoking status							0.490
Never	17		13		16		
Ever	3		4		7		
Family history of tumor							0.021
Absent	14		13		23		
Present	6		4		0		
Tumor diameter							< 0.001
0-10mm	9		3		0		
10-20mm	8		13		12		
20-30mm	3		1		11		
Tumor location							0.012
RUL	8		5		12		
RML	0		3		4		
RLL	2		0		3		
LUL	4		8		4		
LLL	6		1		0		

Radiological type				< 0.001
pGGN	7	4	0	
SSN	13	13	12	
SN	0	0	11	
Surgical approach				< 0.001
Lobectomy	3	10	20	
Sub-lobectomy	17	7	3	

Supplementary table 5. The reproducibility of scRNA-seq data across nine patient donors through irreproducible discovery rate (IDR) analysis.

Cell type	Cell subtype	Group	Marker gene	Irreproducible Discovery Rate
Epithelial	TM4SF1+ cancer cells (Epi-C0)	AIS	TM4SF1	4.48E-06
	TM4SF1+ cancer cells (Epi-C0)	MIA	TM4SF1	4.06E-05
	TM4SF1+ cancer cells (Epi-C0)	IAC	TM4SF1	0.00097041
	CRABP2+ cancer cells (Epi-C3)	AIS	CRABP2	0.266227814
	CRABP2+ cancer cells (Epi-C3)	MIA	CRABP2	0.090658529
	CRABP2+ cancer cells (Epi-C3)	IAC	CRABP2	0.019398353
	UBE2C+ cancer cells (Epi-C6)	IAC	UBE2C	0.003198881
T/NK	NK (T/NK-C4: GNLY+)	AIS	GNLY	7.77E-16
	NK (T/NK-C4: GNLY+)	MIA	GNLY	2.05E-13
	NK (T/NK-C4: GNLY+)	IAC	GNLY	8.10E-11
	Treg (T/NK-C6: FOXP3+)	AIS	FOXP3	0.000171547
	Treg (T/NK-C6: FOXP3+)	MIA	FOXP3	6.86E-08
	Treg (T/NK-C6: FOXP3+)	IAC	FOXP3	2.87E-10
Myeloid	Mast cells (Mye-C0:	AIS	TPSB2	0

	TPSB2+)			
	Mast cells (Mye-C0: TPSB2+)	MIA	TPSB2	4.97E-06
	Mast cells (Mye-C0: TPSB2+)	IAC	TPSB2	5.62E-05
	Monocytes (Mye-C7: FCN1+)	AIS	FCN1	2.59E-05
	Monocytes (Mye-C7: FCN1+)	MIA	FCN1	0.024205964
	Monocytes (Mye-C7: FCN1+)	IAC	FCN1	0.365352387
B cells	MALT B cells (C4: IGHD+)	AIS	IGHD	0.775010024
	MALT B cells (C4: IGHD+)	IAC	IGHD	0.87832332
	Secretory B cells (C7: GZMB+)	AIS	GZMB	0.055846255
	Secretory B cells (C7: GZMB+)	MIA	GZMB	0.042646208
	Secretory B cells (C7: GZMB+)	IAC	GZMB	0.000380491
Endothelial cells	Lymphatic endothelial cells (End-C4: PDPN+)	AIS	PDPN	1.13E-09
	Lymphatic endothelial cells (End-C4: PDPN+)	MIA	PDPN	1.15E-08
	Lymphatic endothelial cells (End-C4: PDPN+)	IAC	PDPN	0
Fibroblast	Cancer-associated fibroblast (Fib-C1)	AIS	FAP	0.000481336
	Cancer-associated fibroblast (Fib-C1)	MIA	FAP	2.74E-07

	Cancer-associated fibroblast (Fib-C1)	IAC	FAP	0.001128006

Supplementary table 6. Ligands and receptors of TGF-β pathway.

Receptor type	Ligands
Type Ⅰ receptor	
ALK1/ACVRL1	TGF-β，BMP9 and BMP10
ALK2/ACVR1	BMPs and GDFs
ALK3/BMPR1A	BMPs
ALK4/ACVR1B	Activins,GDF8/myostatin and GDF11
ALK5/TGFBR1	TGF-βs，GDF/myostatin and GDF11
ALK6/BMPR1B	BMPs
ALK7/ACVR1C	BMP16/nodal
Type Ⅱ receptor	
TGFBR2/TBR Ⅱ	TGF-βs
BMPR2/BMPR Ⅱ	BMPs and GDFs
ACVR2/ActR Ⅱ A	Activins, BMPs and GDFs
ACVR2B/ActR Ⅱ B	Activins, BMPs and GDFs and BMP16/nodal
AMHR2/AMHR Ⅱ	MIF
Type Ⅲ receptor	
Betaglycan	TGF-β1，TGF-β2，TGF-β3，Activin-A,BMP2,BMP4,BMP7 and GDF5
Endoglin	TGF-β1，TGF-β3，Activin-A，BMP2，BMP7 and BMP9

Supplementary table 7. Up-regulated genes in response to TGF-β pathway.

Genes	Gene names
	APC，ARID4B，BCAR3，BMP2，BMPR1A，BMPR2，CDH1，CDK9，CDKN1C，CTNNB1，ENG，FKBP1A，FNTA，FURIN，HDAC1，HIPK2，ID1，ID2，ID3，IFNGR2，JUNB，KLF10，LEFTY2，LTBP2，MAP3K7，NCOR2，NOG，PMEPA1，PPM1A，PPP1CA，PPP1R15A，RAB31，RHOA，SERPINE1，SKI，SKIL，SLC20A1，SMAD1，SMAD3，SMAD6，SMAD7，SMURF1，SMURF2，SPTBN1，TGFB1，TGFBR1，TGIF1，THBS1，TJP1，TRIM33，UBE2D3，WWTR1，XIAP

Supplementary table 8. The reproducibility of ST data across six patient donors through irreproducible discovery rate (IDR) analysis.

Cell type	Cell subtype	Group	Marker gene	Irreproducible Discovery Rate
Cancer cells	TM4SF1+ cancer cells (Epi-C0)	AIS	TM4SF1	0.001465066
	TM4SF1+ cancer	MIA	TM4SF1	0.984152457

	cells (Epi-C0)			
	TM4SF1+ cancer cells (Epi-C0)	IAC	TM4SF1	0.01611888
	CRABP2+ cancer cells (Epi-C3)	AIS	CRABP2	0.874261321
	CRABP2+ cancer cells (Epi-C3)	MIA	CRABP2	0.920942293
	CRABP2+ cancer cells (Epi-C3)	IAC	CRABP2	0.478043675
	UBE2C+ cancer cells (Epi-C6)	AIS	UBE2C	0.8686264
	UBE2C+ cancer cells (Epi-C6)	MIA	UBE2C	0.968448944
	UBE2C+ cancer cells (Epi-C6)	IAC	UBE2C	0.62342401
Lymph	T/NK	AIS	CD3D	0.780143153
	T/NK	MIA	CD3D	0.926281547
	T/NK	IAC	CD3D	0.409710723
	B cells	AIS	CD79A	0.3044021
	B cells	MIA	CD79A	0.882320403
	B cells	IAC	CD79A	0.495937474
Stromal	Endothelial cells	AIS	CLDN5	0.93870687
	Endothelial cells	MIA	CLDN5	0.79757546
	Endothelial cells	IAC	CLDN5	0.851591735
	Fibroblast	AIS	COL1A1	0.021762812
	Fibroblast	MIA	COL1A1	0.780095108
	Fibroblast	IAC	COL1A1	0.254921785
Epithelial cells	AT1	AIS	AGER	5.59E-08
	AT1	MIA	AGER	0.003351366
	AT1	IAC	AGER	0.001500678
	AT2	AIS	SFTPC	6.76E-08
	AT2	MIA	SFTPC	0.013318399
	AT2	IAC	SFTPC	2.37E-05

Supplementary figures

Supplementary Fig.1. Clinical characteristics and cell type identification of LUAD within three pathological stages, related with Fig. 1.

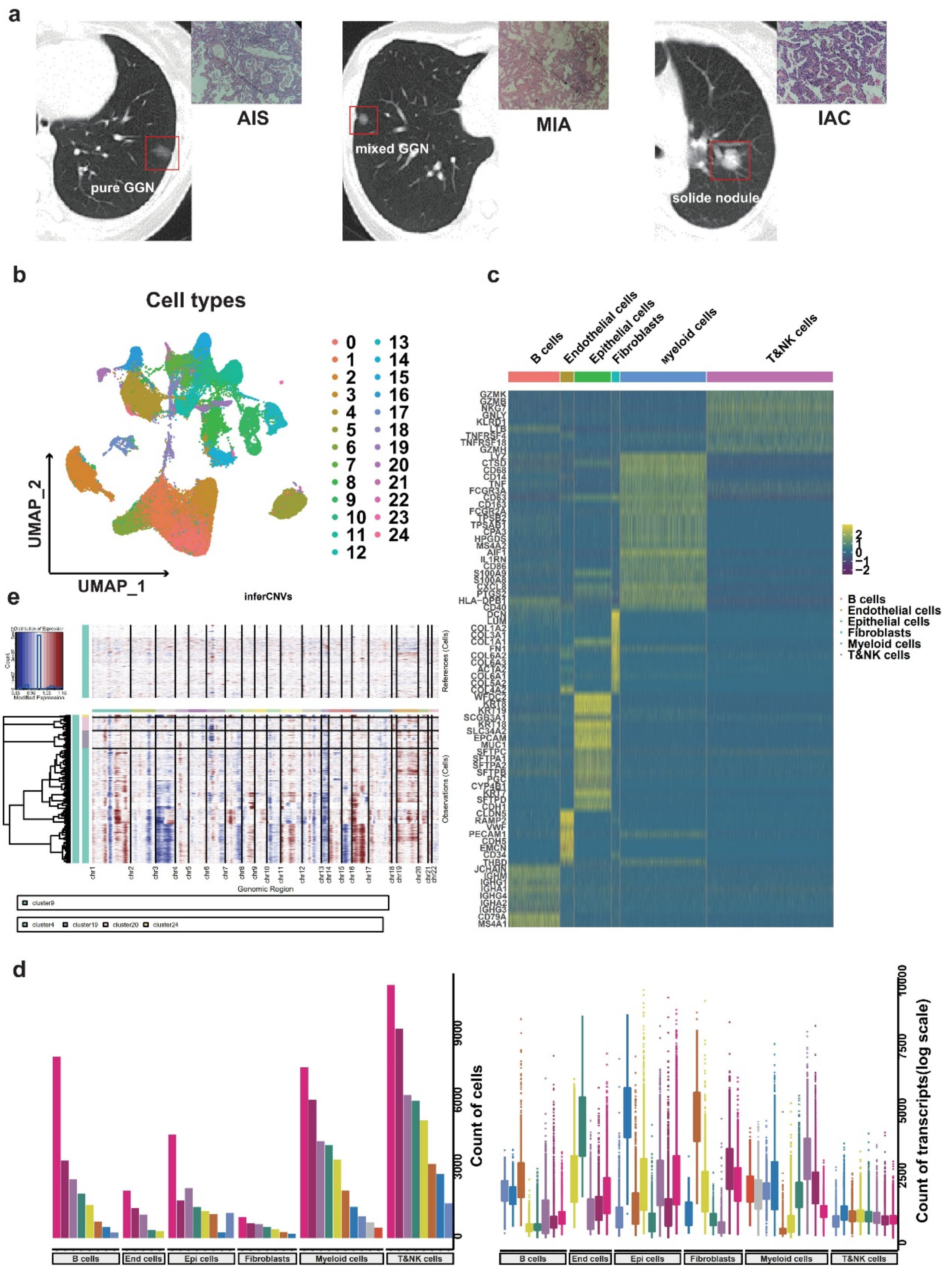

(a) Radiological and pathological features of enrolled cases (left panel: AIS represented by TD8, middle panel: MIA represented by TD3, right panel: IAC represented by TD1); (b) UMAP plot exhibit 25 cell type clusters with high confidence from all nine patients by scRNA-seq; (c) Heat map exhibit marker genes of six dominant cell types from scRNA-seq, the details of marker genes were listed in Table 4.d; (d) Quantity display of number cell (by histogram, left panel) and transcripts (by box diagram, right panel) of each subcluster from all patients in

scRNA-seq data; (e) CNV atlas of cancer cell and normal epithelial cells in LUAD by scRNA-seq. Cluster 4 and Cluster 24 are identified as cancer cells, Cluster 19 and Cluster 20 are identified as normal epithelial cells.

Supplementary Fig.2. Duplicate results for cell type clustering.

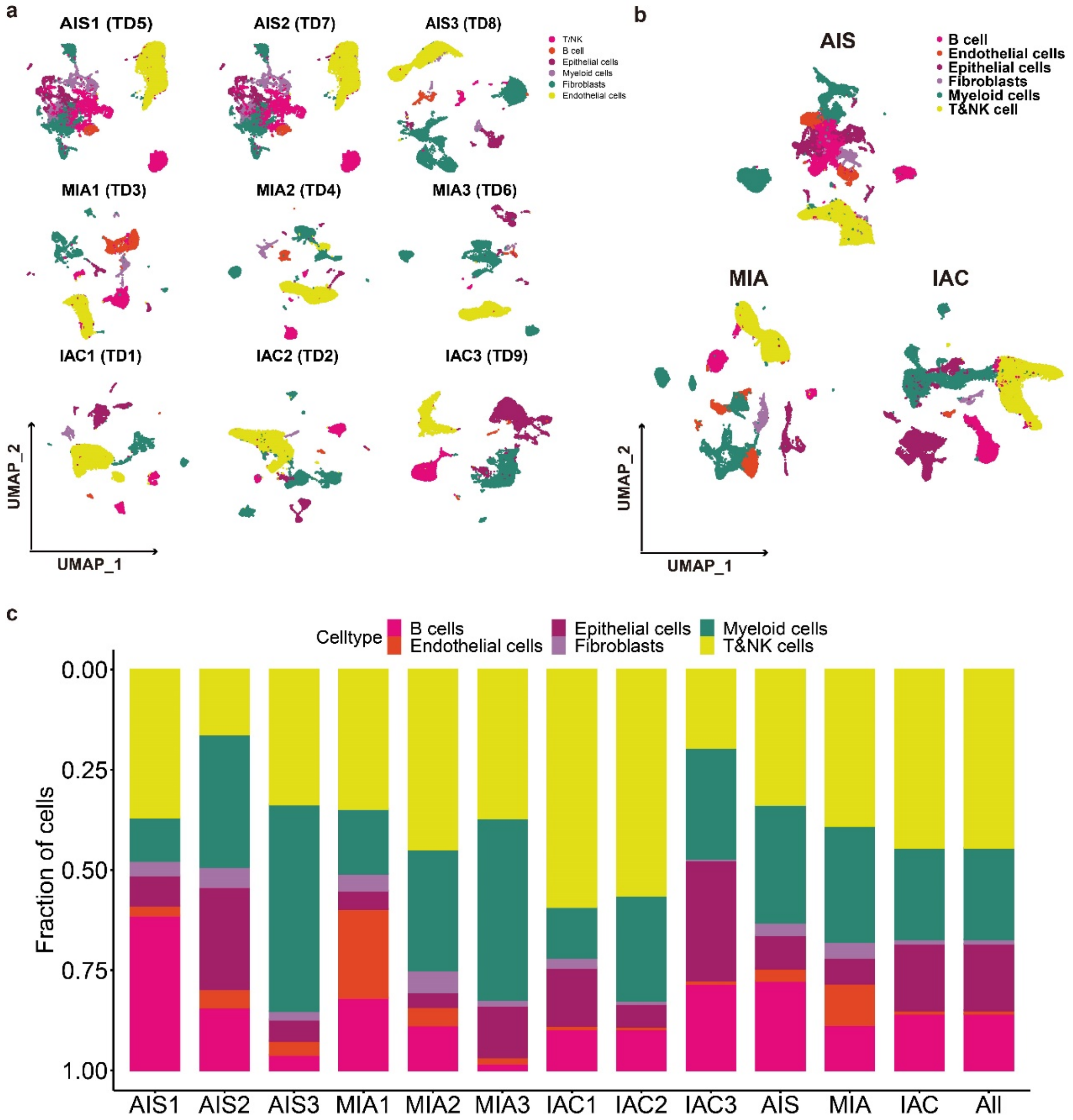

(a) Cell type clustering for each patient in all nine patients; (b) Cell type clustering in three stages of LUAD; (c) Distribution of major cell types in three stages and in each patient of LUAD.

Supplementary Fig.3. Correlation analysis of cellular components from scRNA-seq data.

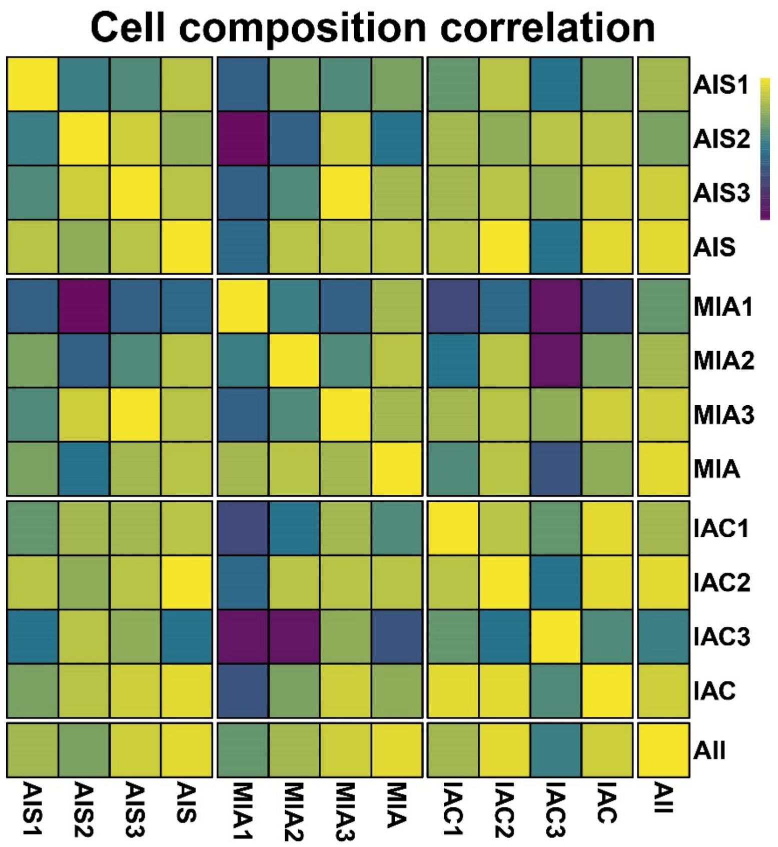

Supplementary Fig.4. Polyclonal origins of cancer cells, related with Fig. 2.

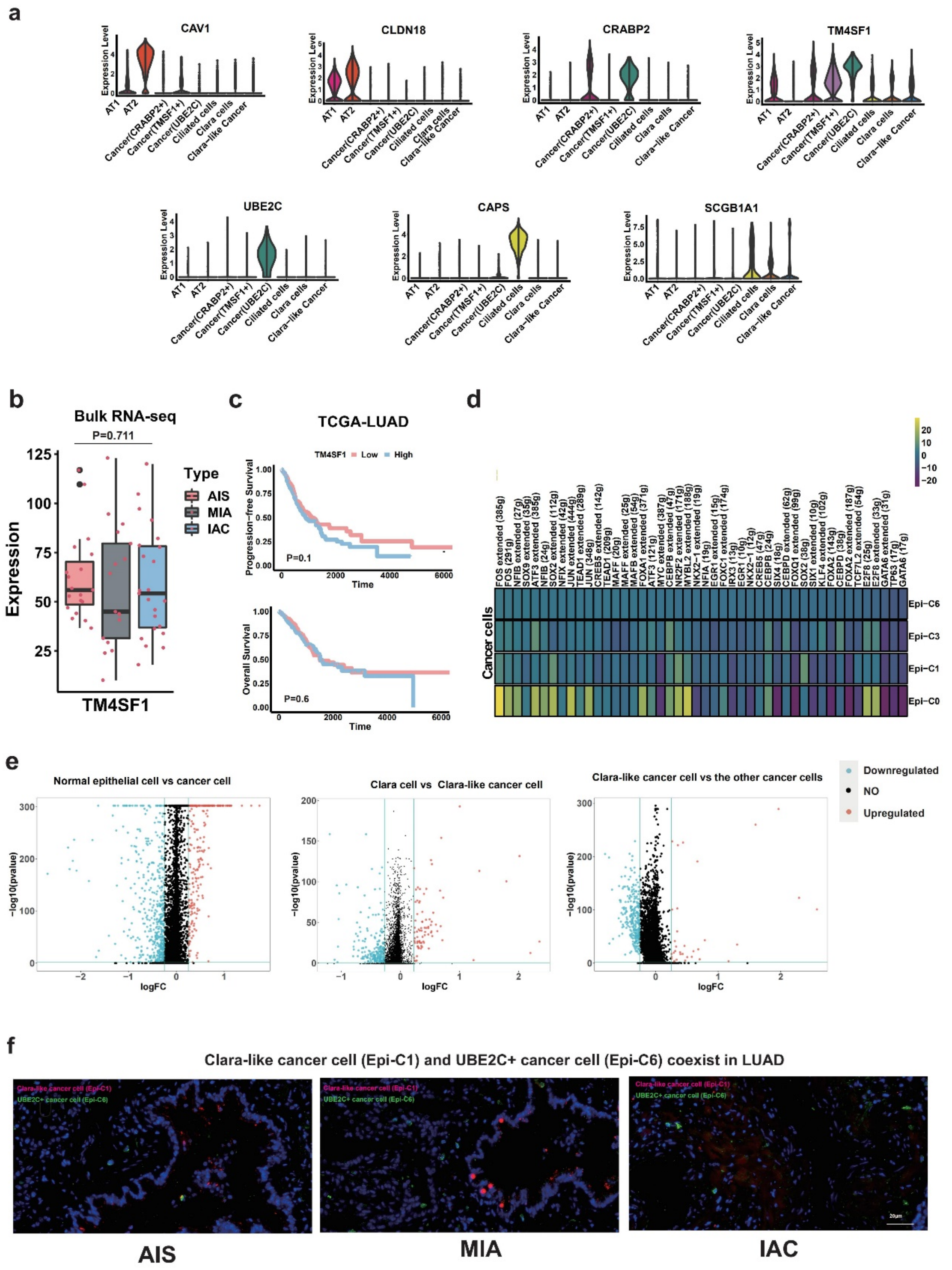

(a) Violin diagram reveal marker genes of four sub-clusters of normal epithelial cells and four sub-clusters of cancer cells. SCGB1A1 is common marker gene of Clara cell and Clara cell-like cancer cell; (b) Bulk RNA-seq validated that the expression level of the TM4SF1 gene increased in the process of cancer invasion; (c) Survival analysis of different TYM4SF1 expression levels in lung adenocarcinoma, from TCGA database; (d) Heat map of the SCENIC results showing the area under the curve (AUC) (enrichment score) for the regulation of TF expression for each kind of tumor

cell sub-cluster; (e) Volcano maps show comparisons of DEGs of Clara-like cancer cells and the other cancer cells from scRNA-seq data. The other normal epithelia cells (Epi-C2, Epi-C4, Epi-C5) vs the other cancer cells (Epi-C0, Epi-C3, Epi-C6); Clara cells (Epi-C7) vs Clara-like cancer cells (Epi-C1), Clara-like cancer cells (Epi-C1) vs the other cancer cells (Epi-C0, Epi-C3, Epi-C6). Blue represent downregulated DEGs, black represent no DEGs, red represent upregulated DEGs; (f) IF staining exhibit coexistence of Clara-like cancer (Epi-C1) and UBE2C (Epi-C6) with three pathological stages in LUAD. Red: Clara-like cancer (Epi-C1); Green: UBE2C (Epi-C6).

Supplementary Fig.5. Decrease of MALT B cells (B-C4: IGHD) drive invasion of LUAD, related with Fig. 4.

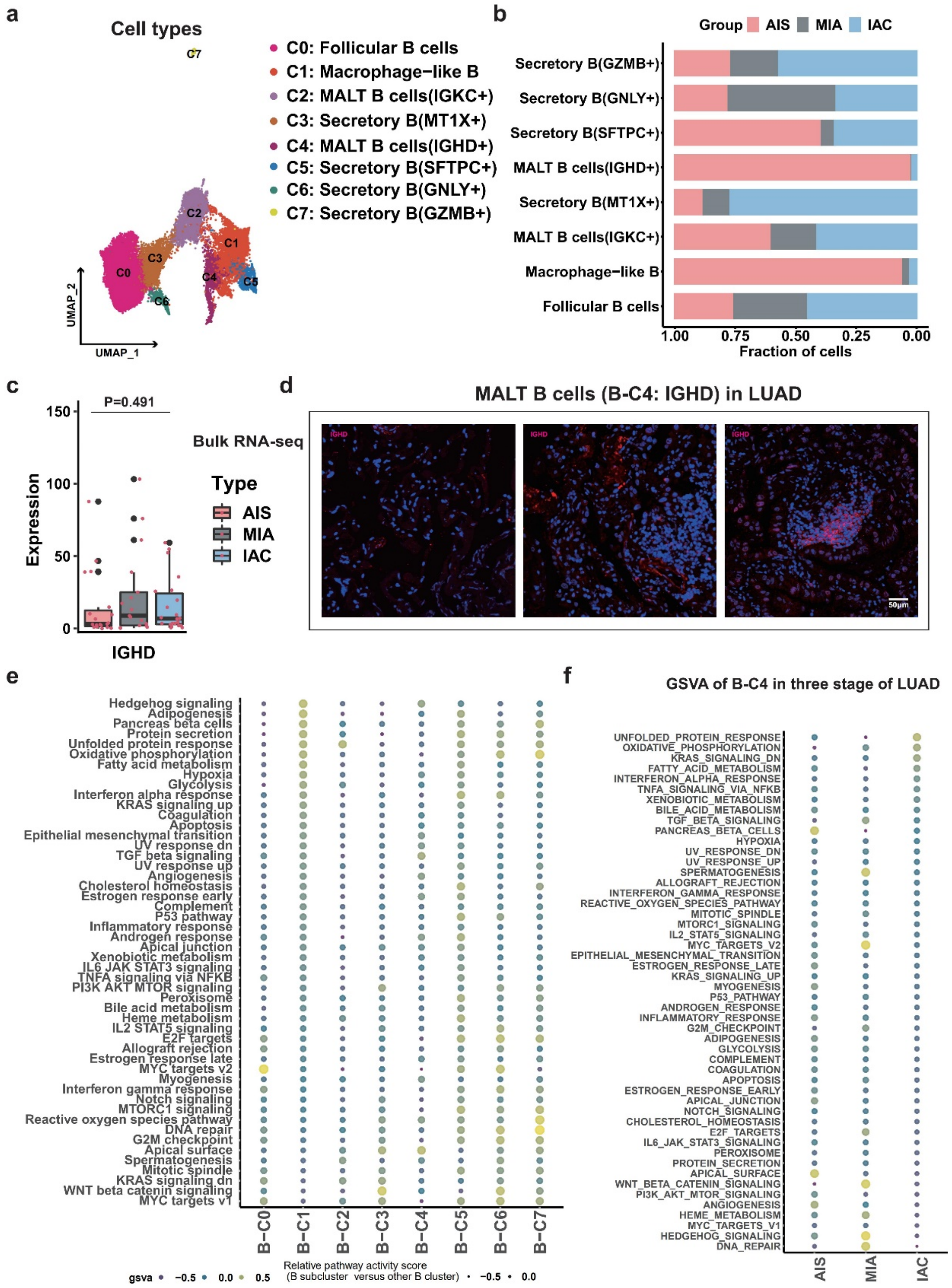

(a) U-MAP of scRNA-seq exhibit eight sub-clusters of B cells from 18,467 cells; (b) Proportions of eight sub-clusters of B cells distributing in three pathological stages of LUAD; (c) Bulk RNA-seq confirm that IGHD expression levels in three stages of LUAD; (d) IF validate that protein expression level of IGHD gradually decreased in the process of cancer invasion in LUAD; (e) Bubble diagram of GSVA exhibit the pathway activity score in each subtype of B cell; (f) Heatmap of GSVA results

showing distinct signaling pathways of MALT B cells (B-C4: IGHD) in three stages of lung adenocarcinoma

Supplementary Fig.6. Decrease of lymphatic endothelial cells (End-C4: PDPN) mediates the invasion of LUAD, related with Fig. 4.

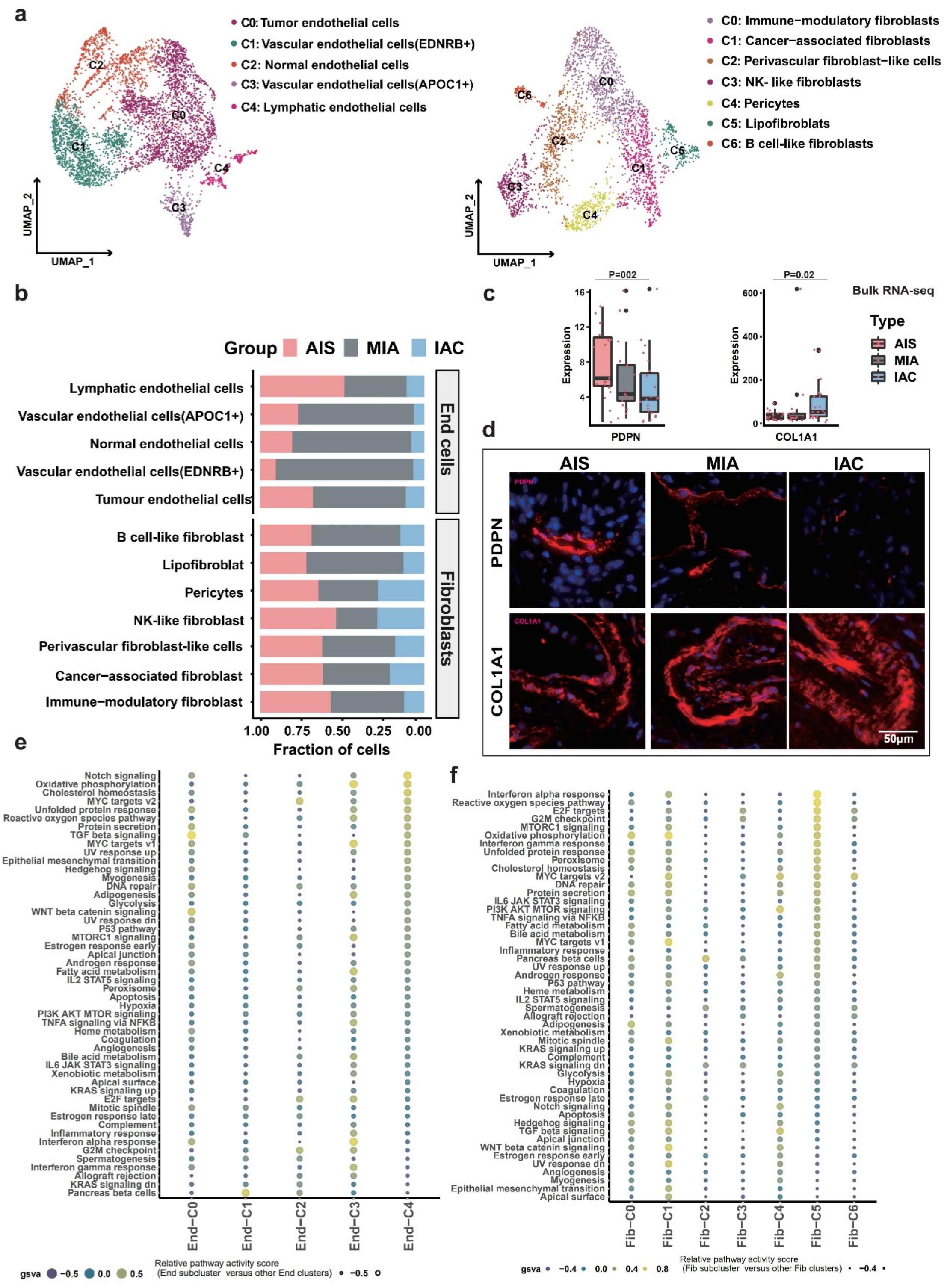

(a) UMAP of scRNA-seq exhibits sub-clusters of endothelial cells (left panel, 4,739

cells, 5 cell subtypes) and fibroblasts (right panel, 2,922 cells, 7 cell subtypes); (b) Proportions of five sub-clusters of endothelial cells (top) and seven sub-clusters of fibroblasts (bottom) distributed in three pathological stages of LUAD; (c) Bulk RNA-seq confirms that the expression level of PDPN gene (marker gene of lymphatic endothelium) gradually decreased from AIS to MIA and then to IAC (left panel), yet COL1A1 gene (marker gene of fibroblasts) show enrich in MIA; (d) IF validate that protein expression level of PDPN gene gradually decreased in the process of cancer invasion in LUAD, however COL1A1 gene show reverse trend; (e) Bubble diagram of GSVA exhibit the pathway activity score in each type of endothelial cell sub-clusters; (f) Bubble diagram of GSVA exhibit the pathway activity score in each type of fibroblasts sub-clusters.

Supplementary Fig.7. The reproducibility of gene expression for each major cell type through Wilcoxon on test for each individual patient.

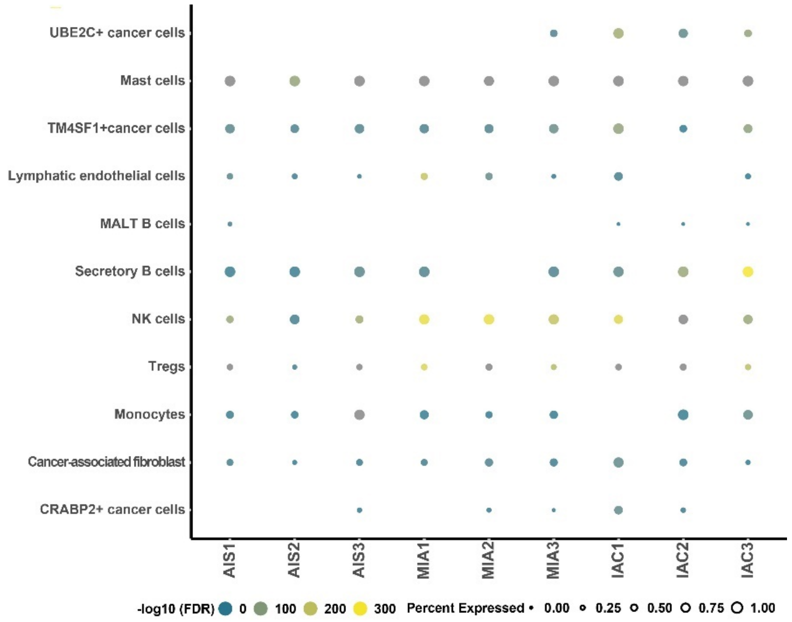

Supplementary Fig.8. Prediction of the interaction of receptors and ligands in cancer cells and TME cells.

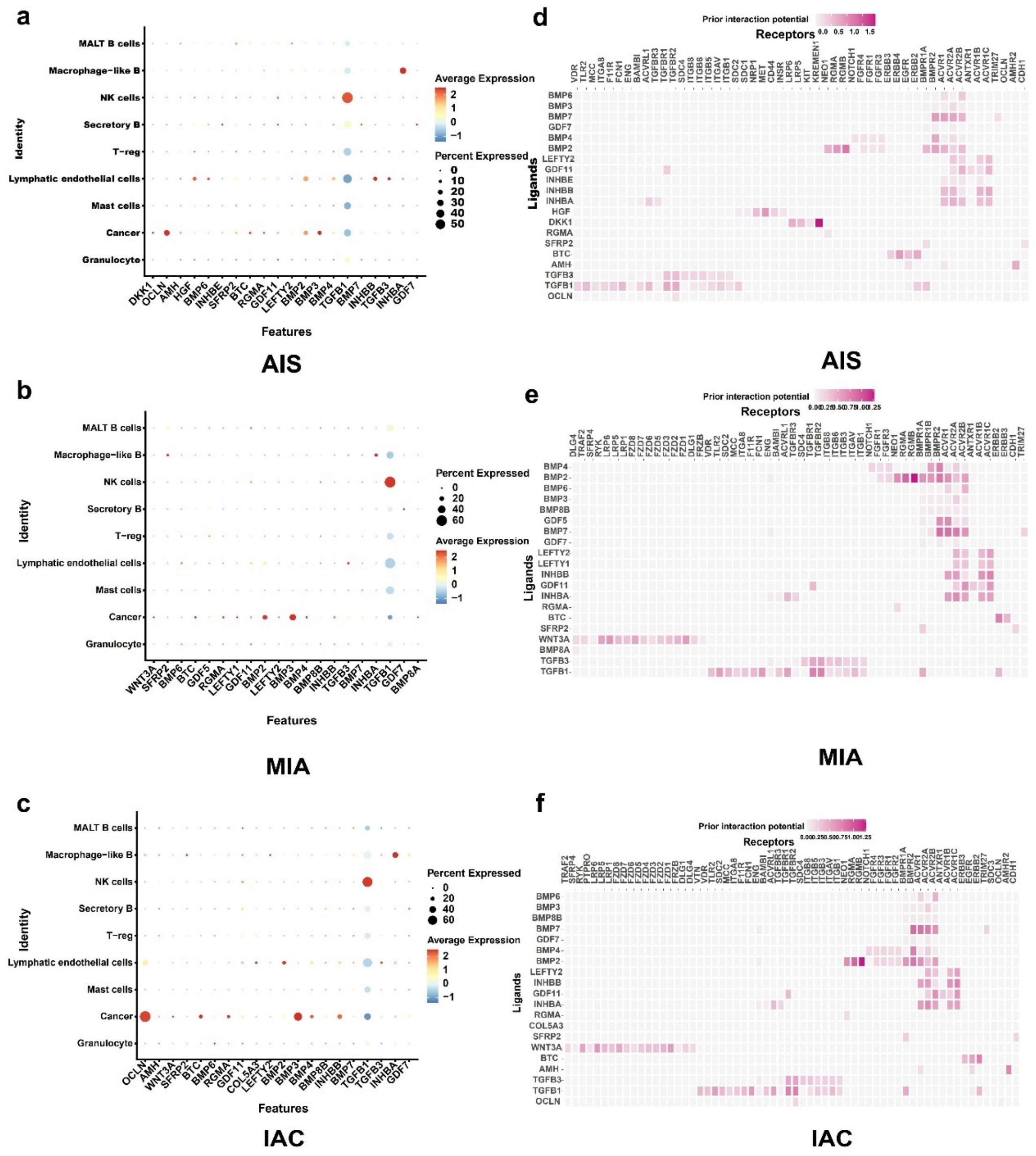

Top predicted ligand-receptor interactions presented between cancer cells and TME cells in AIS (a), MIA (b) and IAC (c). Top ligand-receptor interactions predicted by NicheNet in AIS (d), MIA (e) and IAC (f).

Supplementary Fig.9. Prediction of signaling pathways in cancer cells and TME cells.

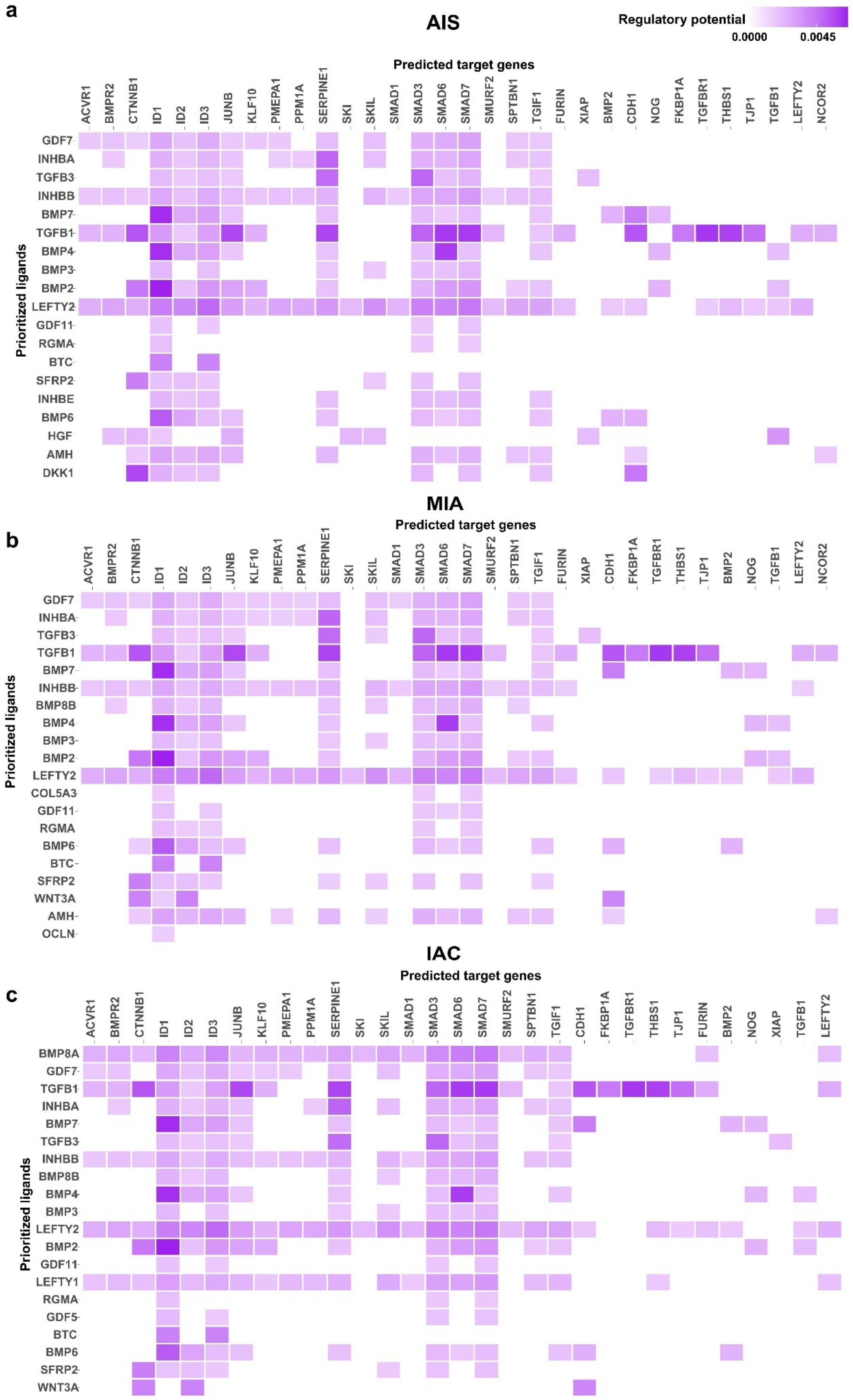
a
AIS
Regulatory potential
0.0000
0.0045
Predicted target genes
ACVR1 BMPR2 CTNNB1 ID1 ID2 ID3 JUNB KLF10 PMEPA1 PPM1A SERPINE1 SKI SKIL SMAD1 SMAD3 SMAD6 SMAD7 SMURF2 SPTBN1 TGIF1 FURIN XIAP BMP2 CDH1 NOG FKBP1A TGFBR1 THBS1 TJP1 TGFB1 LEFTY2 NCOR2
Prioritized ligands
GDF7 INHBA TGFB3 INHBB BMP7 TGFB1 BMP4 BMP3 BMP2 LEFTY2 GDF11 RGMA BTC SFRP2 INHBE BMP6 HGF AMH DKK1
MIA
Predicted target genes
b
ACVR1 BMPR2 CTNNB1 ID1 ID2 ID3 JUNB KLF10 PMEPA1 PPM1A SERPINE1 SKI SKIL SMAD1 SMAD3 SMAD6 SMAD7 SMURF2 SPTBN1 TGIF1 FURIN XIAP CDH1 FKBP1A TGFBR1 THBS1 TJP1 BMP2 NOG TGFB1 LEFTY2 NCOR2
Prioritized ligands
GDF7 INHBA TGFB3 TGFB1 BMP7 INHBB BMP8B BMP4 BMP3 BMP2 LEFTY2 COL5A3 GDF11 RGMA BMP6 BTC SFRP2 WNT3A AMH OCLN
IAC
Predicted target genes
c
ACVR1 BMPR2 CTNNB1 ID1 ID2 ID3 JUNB KLF10 PMEPA1 PPM1A SERPINE1 SKI SKIL SMAD1 SMAD3 SMAD6 SMAD7 SMURF2 SPTBN1 TGIF1 CDH1 FKBP1A TGFBR1 THBS1 TJP1 FURIN BMP2 NOG XIAP TGFB1 LEFTY2
Prioritized ligands
BMP8A GDF7 TGFB1 INHBA BMP7 TGFB3 INHBB BMP8B BMP4 BMP3 LEFTY2 BMP2 GDF11 LEFTY1 RGMA GDF5 BTC BMP6 SFRP2 WNT3A

(a) The predicted target genes in AIS are predicted by NicheNet; (b) The predicted target genes in MIA are predicted by NicheNet; (c) The predicted target genes in IAC predicted by NicheNet.

Supplementary Fig.10. Correlation analysis of the number of spots in each region from ST data.

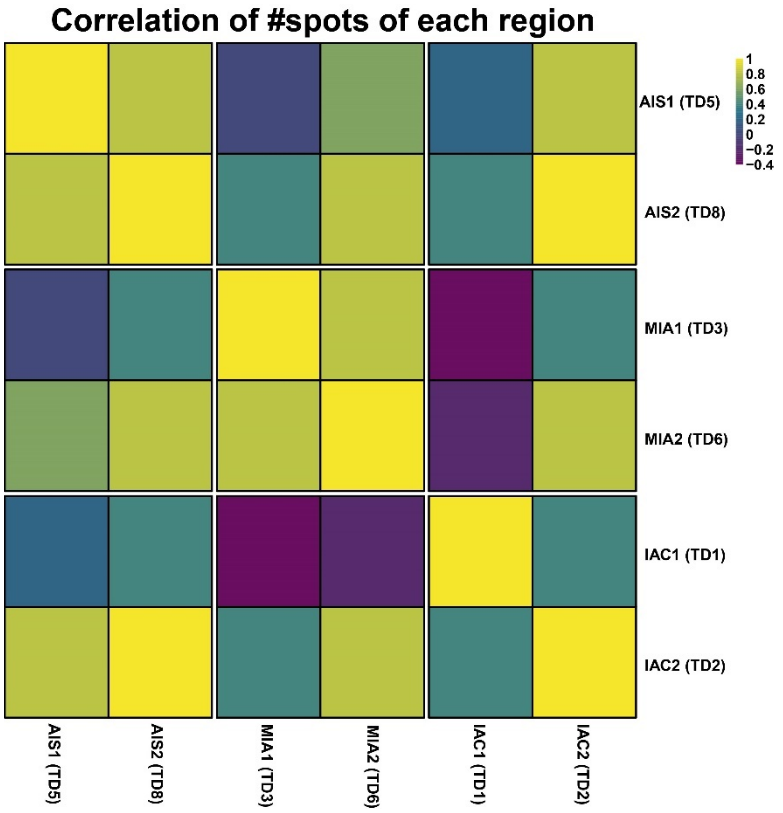

Supplementary Fig.11. Analysis of cellular spatial distribution and integration of single cell data in three pathological stages of LUAD, related with Fig. 6.

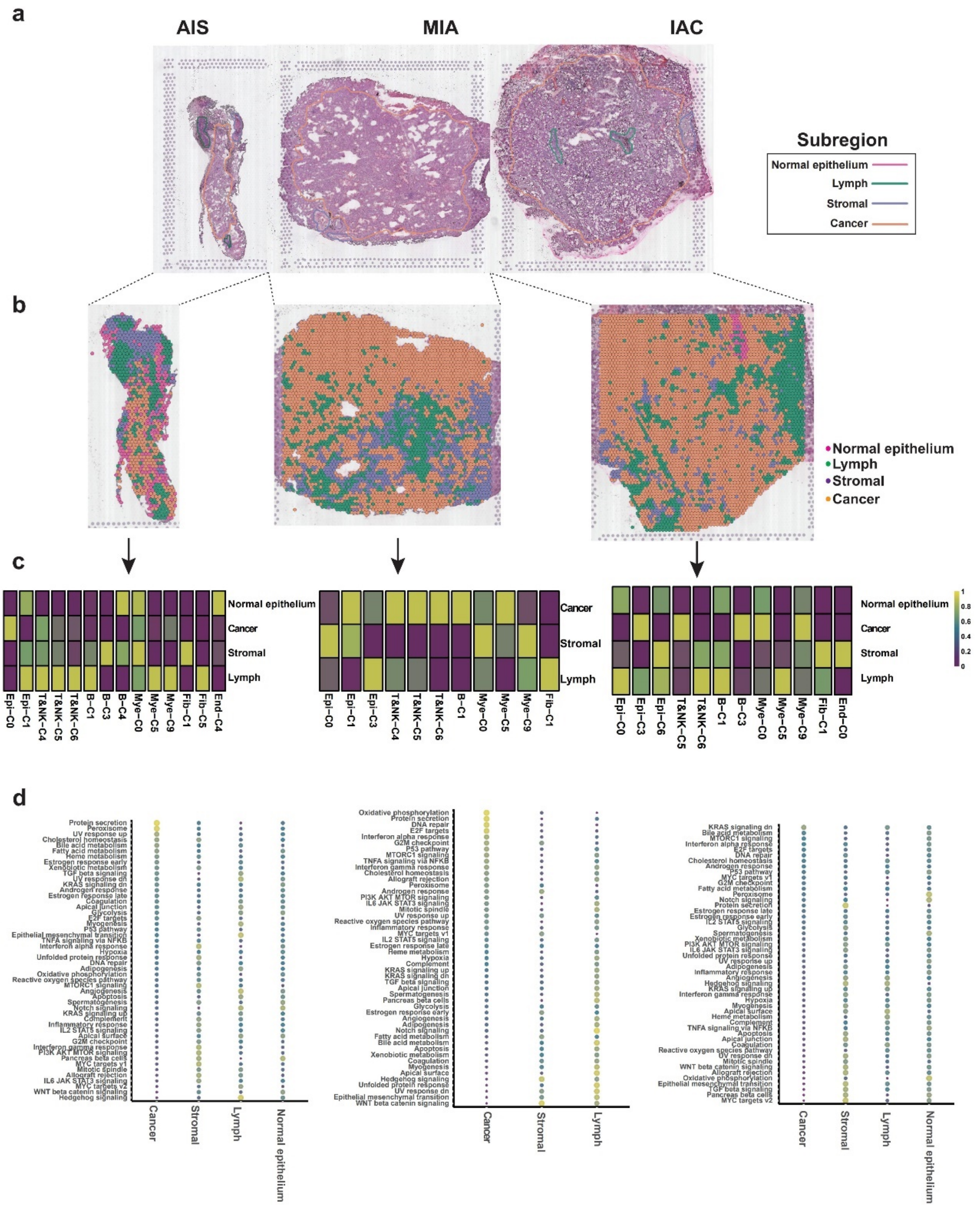

(a) HE staining exhibits the histological regions of AIS (left panel, TD5), MIA (middle panel, TD6) and IAC (right panel, TD2). Pink label: normal epithelium, green label: lymph, purple label: stromal, yellow label: cancer; (b) The spatial subregions of three pathological stages of LUAD annotated by ST; (c) Hyper-geometric maps of all certain cell types identified by scRNA-seq and regions defined by ST, yellow indicates high overlap enrichment; purple indicates low overlap enrichment; (d) Bubble diagram of GSVA exhibit the pathway activity score in each type of spatial

region.

Supplementary Fig.12. Cell types of each spatial spot for three stages of LUAD (TD8, TD3, and TD1) revealed by RCTD.

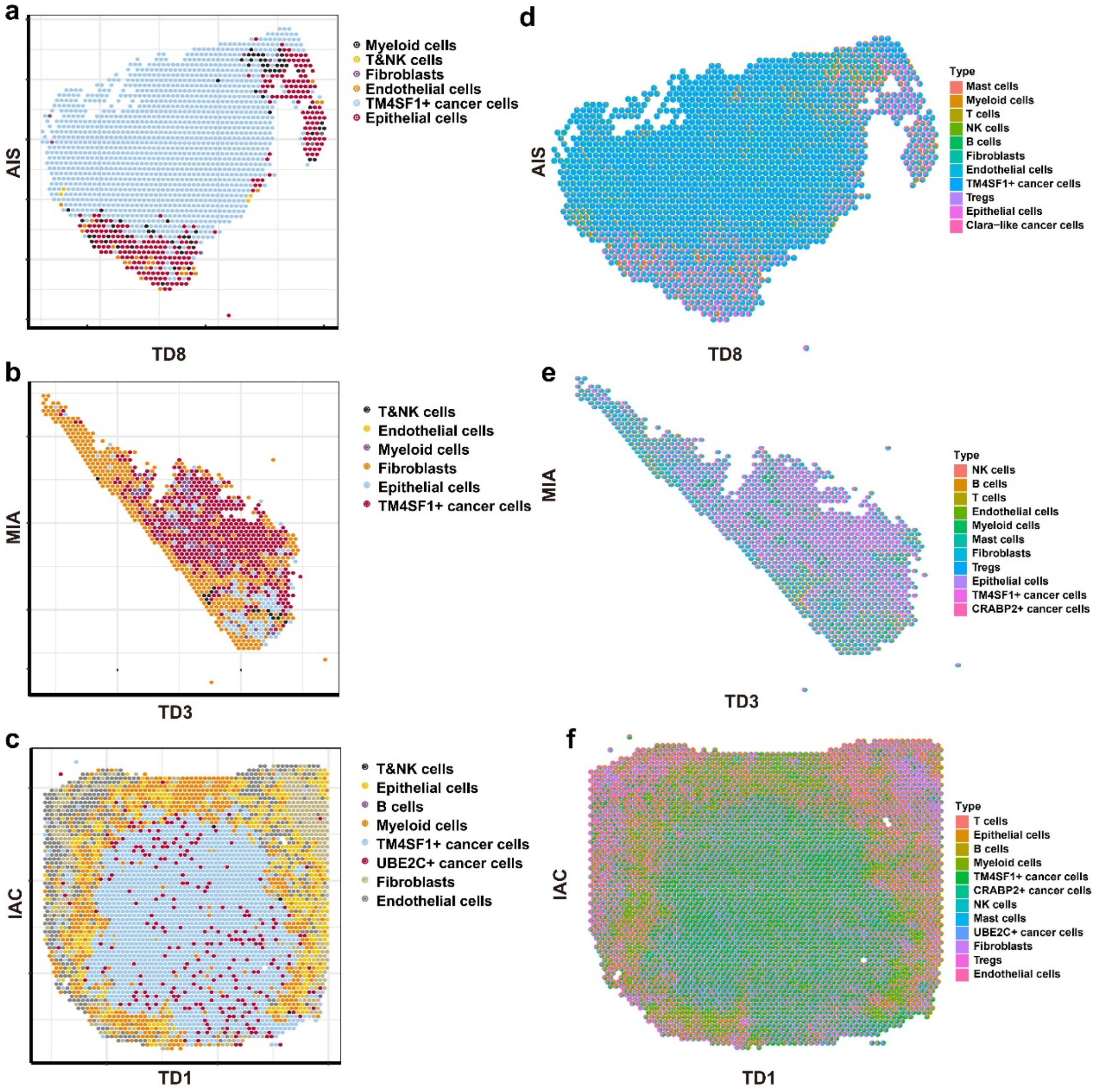

Major cell types in each spatial spot for AIS (TD8, a), MIA (TD 3, b), and IAC (TD 1, c); Pie chart of cell types in each spatial spot for AIS (d), MIA (e), and IAC (f).

Supplementary Fig.13. Cell types of each spatial spot for three stages of LUAD (TD5, TD6, and TD2) revealed by RCTD.

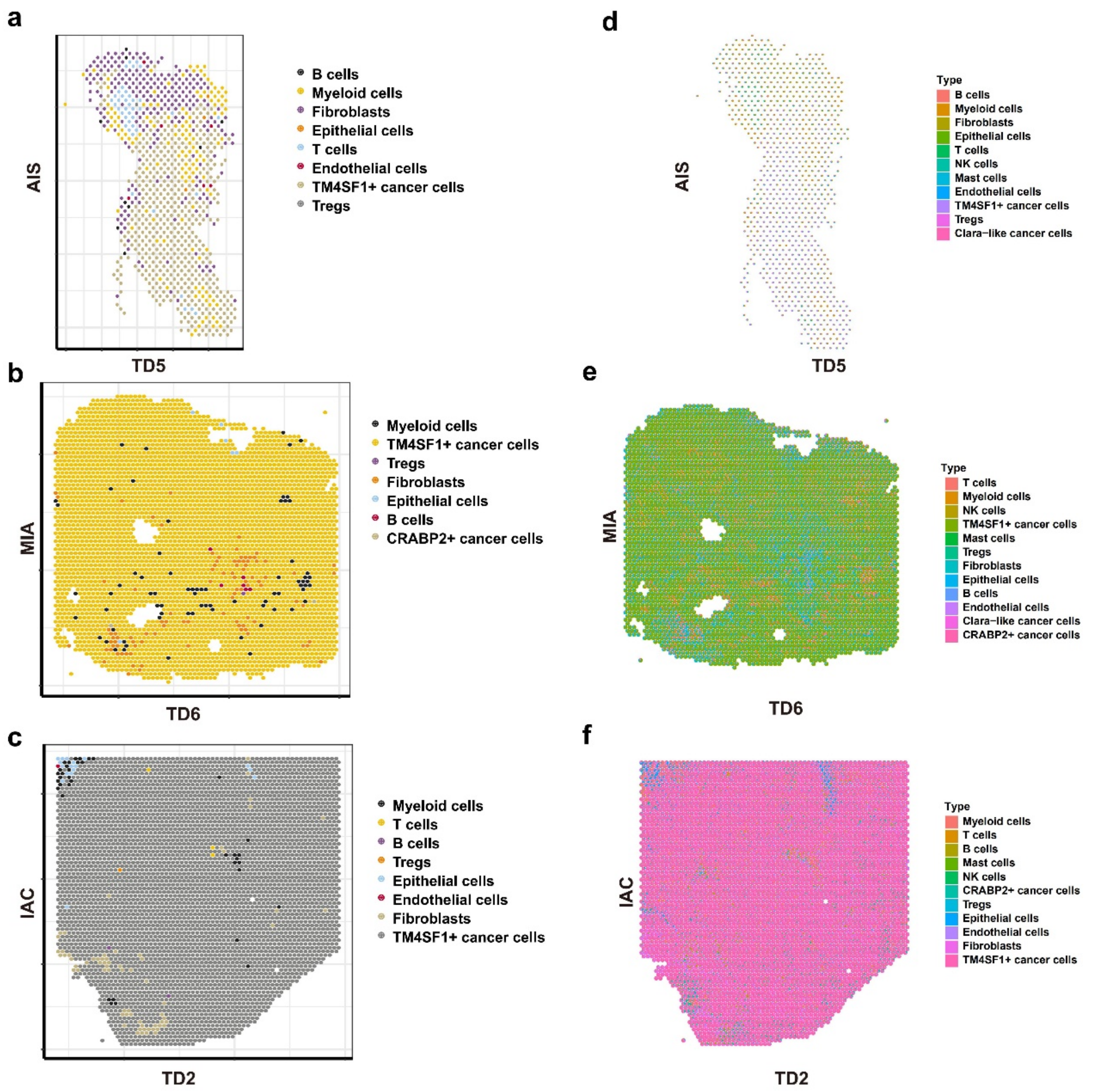

Major cell types in each spatial spot for AIS (TD5, a), MIA (TD 6, b), and IAC (TD 2, c); Pie chart of cell types in each spatial spot for AIS (d), MIA (e), and IAC (f).

Supplementary Fig.14. Prediction of major cell types in LUAD based on each spatial spot by using Cell-ID.

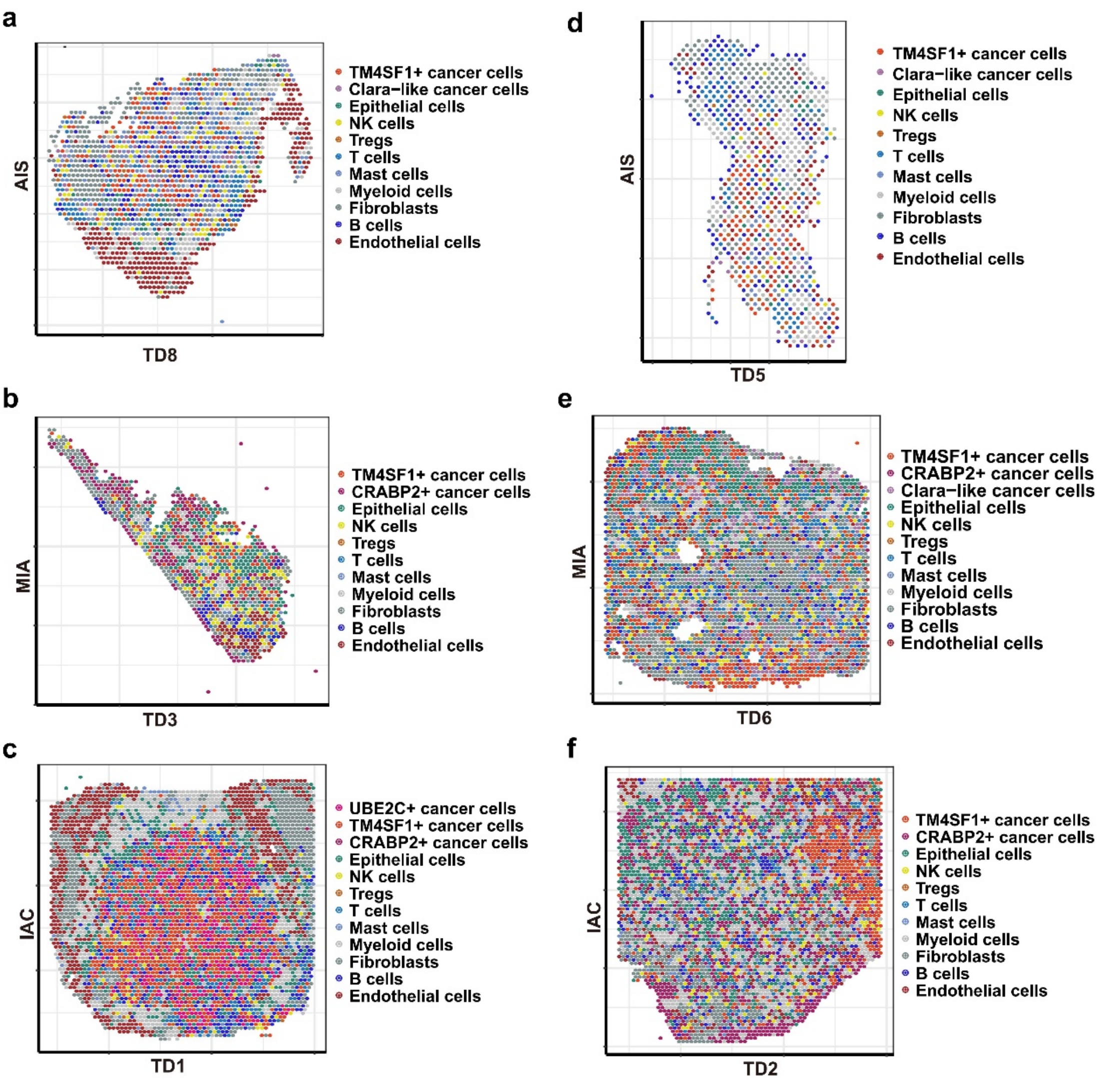

Cell types in the spatial spot of AIS (a, d), MIA (b, e) and IAC (c, f).

Supplementary Fig.15. Spatial differentiation trajectories of LUAD.

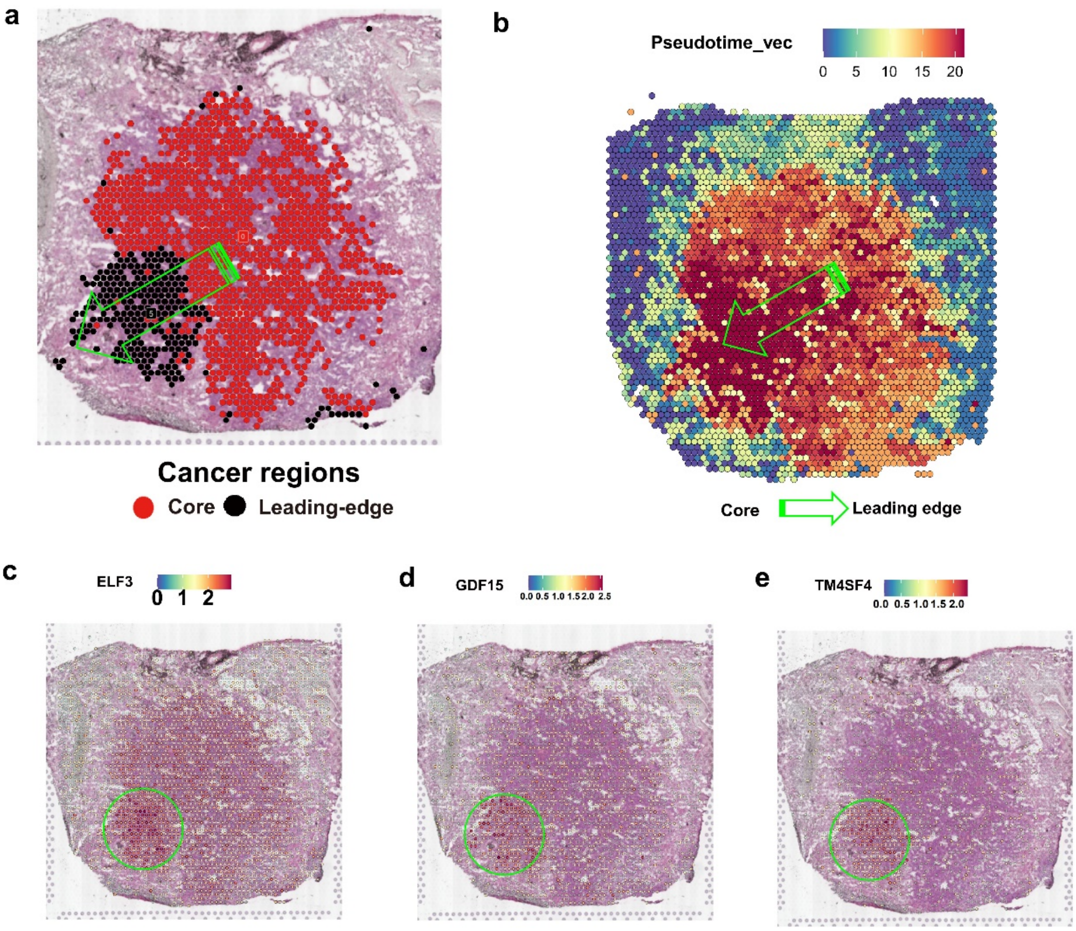

(a) ST analysis is used to sub-regionalize the cancer region; (b) The pseudo-time analysis of different regions (edge and core) of the tumor using SPAPA; Key marker gene of the leading edge were ELF3(c), GDF15 (d), and TM4SF4 (e).

Supplementary Fig.16. Spatial transcriptome and re-clustering of cancer cells， related with Fig. 7.

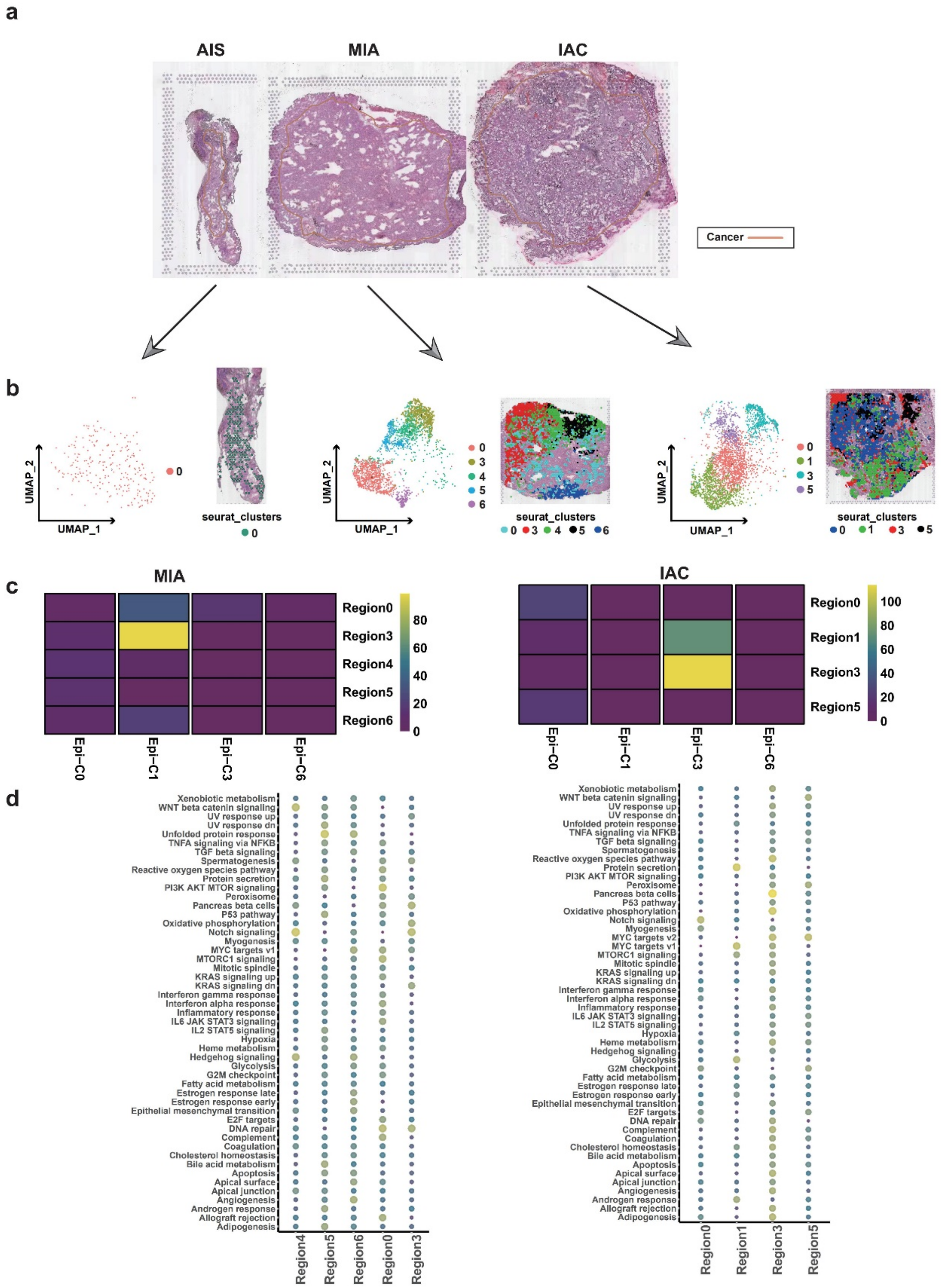

(a) HE staining exhibit the histological cancer subregions of AIS (left panel, TD5), MIA (middle panel, TD6) and IAC (right panel, TD2); (b) UMAP plot exhibit varieties of cancer subregions in HE-stained cancer region by ST; (c) Heat maps showing four cancer subclusters (Epi-C0, Epi-C1, Epi-C3, Epi-C6) obtained upon hyper-geometric distribution analysis of all specific cell types identified by scRNA-seq analysis and regions defined by ST analysis (left panel: MIA represented by TD6, right panel: IAC represented by TD2); (d) Bubble diagram of GSVA exhibit

the pathway activity score in each type of spatial subregion (left panel: MIA represented by TD6, right panel: IAC represented by TD2).
